# Double layer charging driven carbon dioxide adsorption limits the rate of electrochemical carbon dioxide reduction on Gold

**DOI:** 10.1038/s41467-019-13777-z

**Published:** 2020-01-07

**Authors:** Stefan Ringe, Carlos G. Morales-Guio, Leanne D. Chen, Meredith Fields, Thomas F. Jaramillo, Christopher Hahn, Karen Chan

**Affiliations:** 1 SUNCAT Center for Interface Science and Catalysis, Department of Chemical Engineering, Stanford University, Stanford, CA 94305 USA; 2 SUNCAT Center for Interface Science and Catalysis, SLAC National Accelerator Laboratory, Menlo Park, CA 94025 USA; 3 Department of Chemical and Biomolecular Engineering, University of California, Los Angeles, CA 90095 USA; 4 Department of Chemistry, University of Guelph, Guelph, ON N1G 2W1 Canada; 5 CatTheory Center, Department of Physics, Technical University of Denmark, Kongens Lyngby, 2800 Denmark

**Keywords:** Catalysis, Chemical engineering, Carbon capture and storage, Chemical engineering

## Abstract

Electrochemical CO$_{2}$ reduction is a potential route to the sustainable production of valuable fuels and chemicals. Here, we perform CO$_{2}$ reduction experiments on Gold at neutral to acidic pH values to elucidate the long-standing controversy surrounding the rate-limiting step. We find the CO production rate to be invariant with pH on a Standard Hydrogen Electrode scale and conclude that it is limited by the CO$_{2}$ adsorption step. We present a new multi-scale modeling scheme that integrates ab initio reaction kinetics with mass transport simulations, explicitly considering the charged electric double layer. The model reproduces the experimental CO polarization curve and reveals the rate-limiting step to be *COOH to *CO at low overpotentials, CO$_{2}$ adsorption at intermediate ones, and CO$_{2}$ mass transport at high overpotentials. Finally, we show the Tafel slope to arise from the electrostatic interaction between the dipole of *CO$_{2}$ and the interfacial field. This work highlights the importance of surface charging for electrochemical kinetics and mass transport.

## Abstract (web summary)

Electrochemical CO_2_ reduction is a potential route to the sustainable production of valuable fuels and chemicals. In this joint experimental-theoretical work, the authors address the issue of the rate-limiting step on Gold and present insights from multi-scale simulations into the importance of the electric double layer on reaction kinetics and mass transport.

## Introduction

In light of the urgent need to mitigate climate change and the wide interest in carbon dioxide (CO$_{2}$) sequestration and conversion, CO$_{2}$ electroreduction (CO$_{2}$R) has emerged as an attractive prospect toward establishing a sustainable carbon cycle. When coupled to renewable electricity, CO$_{2}$R would allow for the storage of intermittent renewable resources at ambient pressures and temperatures, produce fuels, and chemicals, all the while reducing CO$_{2}$ emissions^1^. Gold-based materials are the most active and selective catalysts for the production of CO, a critical component of syngas^2^. Understanding the reaction mechanism on Gold-based catalysts is therefore a critical step toward designing new catalyst materials, and is the focus of the present joint theoretical–experimental study.

While it is generally accepted that CO$_{2}$R to CO proceeds through *COOH and *CO intermediates, the rate-limiting step remains a topic of significant controversy. On the basis of recent Tafel analysis and kinetic isotope effect (KIE) studies, three different reaction steps have been postulated to be rate limiting: electron transfer to CO$_{2}$ and concomitant adsorption^3,4^, proton transfer to *CO$_{2}$ to form *COOH or *COOH to form a protonated *COOH complex^5^, or electron transfer to *COOH to form *CO^6^. In this work, we will discuss the reasons for the controversy and present experimental and theoretical evidence for field-driven CO$_{2}$ adsorption as the limiting step.

Recently, a few attempts have been made to develop kinetic models for CO$_{2}$ reduction to CO based on ab initio reaction energetics, which focus primarily on Ag^7,8^. A previous study has also constructed a kinetic volcano for CO$_{2}$ reduction to CO based on an electrochemical barrier fitted from experiment along with ab initio calculations of reaction thermodynamics^9^. Due to the complexity of the reaction network, Bagger et al. also correlated reaction thermodynamics directly with product selectivity in CO$_{2}$R in a phenomenological manner^10,11^. The theoretical studies suggest different rate-limiting steps, which could arise from the vastly different methodologies and model assumptions, and highlight the need for a new, unified multiscale modeling approach. In addition, recent studies have stressed the importance of mass transport phenomena^12–15^. Attempts have been made to include these in kinetic models^8,12,16^, however, without considering the presence of the charged double layer. The significant impact of the electric double-layer field on the energetics of critical reaction steps has also been suggested by recent studies^7,17–20^. Here, we show that the double layer plays a critical role for both kinetics and mass transport through its impact on the pH and reactant concentrations at the reaction plane.

In what follows, we first present CO$_{2}$R experiments on Gold at acidic pH values similar to a recent study by Varela et al. on single-site catalysts^21^. Where hydronium cations are proton donors, the first CO$_{2}$ adsorption step and the proton-coupled electron transfer (PCET) steps can be clearly distinguished by their pH dependence. We therefore attribute the invariance of the CO production rate to the CO$_{2}$ adsorption step being limiting. We develop a microkinetic model based on surface-charge-dependent ab initio-derived reaction energetics. The kinetics are coupled to a detailed continuum model that integrates, for the first time, the structure of the electric double layer, buffer equilibria, diffusion, and migration. The simulations confirm the CO$_{2}$ adsorption step to be rate limiting. We show the experimental Tafel slope to arise from the potential dependence of the surface-charge density and the corresponding electric double-layer field, which drives the adsorption of CO$_{2}$. At very low overpotentials, the reaction rate is limited by the conversion of *COOH to *CO. We also find that the double-layer charging reduces the local pH at the reaction plane, making CO$_{2}$ adsorption a more likely rate-limiting step than the formation of *COOH. These results together demonstrate the critical role played by the structure of the electric double layer on both the rate of field-driven processes and the local pH, which drives proton transfers in electrochemical energy conversion processes.

## Results

### Reaction mechanism

**Fig. 1:**
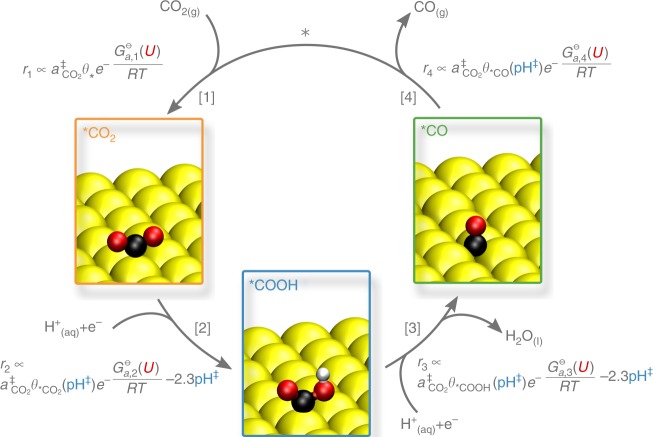
Considered reaction mechanism for CO$_{2}$R on Gold. The gray equations depict the measurable CO production rate ${r}_{{{k}}}$ in the case that the corresponding reaction step $k$ is limiting the overall conversion process, ${a}_{i}^{\ddagger }$ and pH$^{\ddagger }$ depicts species activities and pH at the reaction plane. All equations are given for acidic reaction conditions on an absolute potential reference scale. The coverage of empty sites (${\theta }_{* }$) is roughly pH independent, since it is always close to one on Gold.

Figure 1 shows the commonly discussed reaction mechanism for CO$_{2}$R on Gold electrodes, applied in this work^5,22,23^. The process is initiated by the adsorption of CO$_{2}$. As CO$_{2}$ approaches the surface, it bends, resulting in a stabilization of the lowest unoccupied molecular orbital (LUMO) and a partially negative *CO$_{2}$ intermediate^5,24^, which can be stabilized by the interfacial electric field^7,18–20^. The CO$_{2}$ adsorption step is followed by two proton-coupled electron transfers (PCETs) to yield the *CO intermediate, which readily desorbs due to the weak CO–Au binding energy^25^.

### Experimental evidence for the rate-limiting step

While the mechanism illustrated in Fig. 1 is generally accepted, the rate-limiting step remains controversial. In recent literature, proton-electron transfer to *CO$_{2}$ to form *COOH^5^ and *COOH to form *CO^6^, as well as CO$_{2}$ adsorption with concomitant electron transfer^3,4^ have all been proposed to be limiting.

To elucidate the rate-limiting step, we performed pH-dependent CO$_{2}$R experiments over an acidic to neutral pH range. As shown in the rate expressions in Fig. 1 (cf. also Supplementary Note 1) if CO$_{2}$ adsorption were rate-limiting, the activity would show no pH dependence on an absolute potential reference scale (e.g., on the standard hydrogen electrode—SHE scale), as long as the coverage of empty sites are pH independent. On Gold, experimental and theoretical results, as well as our simulations below, suggest negligible coverages of reaction intermediates^3,26,27^, in which case the relative change of the coverage of free active sites with a change in the pH is negligibly small. In contrast to CO$_{2}$ adsorption, all other reaction steps depend on the pH either directly due to protons being reactants or indirectly via the surface coverage of reactants, suggesting that the pH dependence can be used to distinguish the rate-limiting step.

**Fig. 2:**
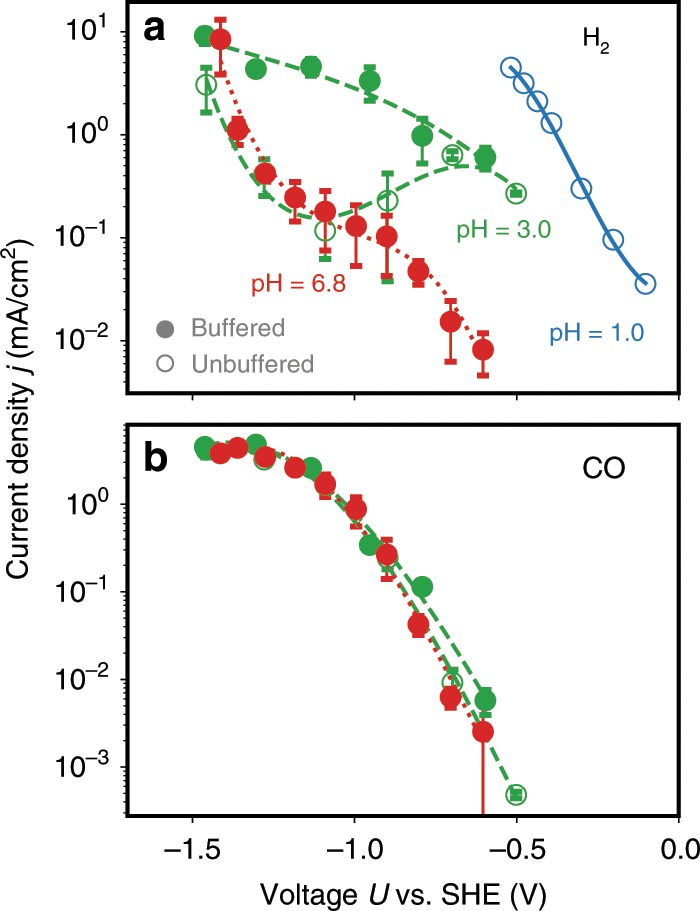
Experimental CO$_{2}$R polarization curves. **a** H$_{2}$ and **b** CO partial current density. The data was obtained from running CO$_{2}$R experiments on a polycrystalline Gold electrode at various bulk pH conditions. The red, green and blue circles refer to bulk pH values of 6.8, 3.0 and 1.0, respectively. Filled circles denote buffered conditions using 0.1 M potassium bicarbonate (KHCO_3_, pH 6.8) and 0.1 M potassium phosphate (KH_2_PO_4_, pH 3.0), while the empty circles refer to unbuffered conditions using 0.1 M potassium perchlorate (KClO_4_, pH 3.0) and 0.1 M perchloric acid (HClO_4_, pH 1.0) as electrolytes. The vertical error bars represent the standard deviation resulting from three separate measurements.

Figure 2 shows the experimentally obtained partial current density for H$_{2}$ (a) and CO (b) production on an SHE scale under the influence of different bulk pH values and buffering conditions (faradaic efficiencies are shown in Supplementary Note 2, in particular Supplementary Figs. 1–4 and Supplementary Tables 1–4). First, a clear shift of the curves with pH is observed in the HER case. Due to the small concentration of protons at pH = 6.8, water would be the dominant proton donor. At pH = 3.0, the proton activity increases significantly, and with it, the HER overpotential drops. Due to the higher acidity of hydronium ions compared with water, they would dominate the proton transfers to adsorbates. Figure 2 also shows the activity of an unbuffered solution. In the HER activity, there is a plateau in the intermediate-potential region, which can be attributed to mass transport limitations of protons^28^. We additionally observed a decrease in the rate of HER in unbuffered systems as a result of an increase in pH over the duration of the experiment and at more negative potentials. The slight increase in pH, which we kept below 0.5 units (reported in the “Methods” section), will be proportional to the total number of charges passed during the experiment. From the Nernst equation, this increase would lead to an estimated decrease in HER rate by 68% in agreement with our experiment. Going to more negative overpotentials, the strong faradaic currents reduce the proton activity at the electrode, until water becomes a more facile proton donor and the current density approaches the one at pH = 6.8 conditions^29^.

CO production was observed under all pH conditions except at pH 1, where only H$_{2}$ could be collected. In contrast to the HER polarization curves, the CO partial current density is invariant with pH, as clearly seen from Fig. 2. This invariance shows that the CO$_{2}$ adsorption step is limiting the reaction rate, since otherwise the overpotential should also shift with pH, similar to the HER current densities. Also, the observed lack of influence of the presence of buffer at pH 3.0 could be seen as another hint for a non-proton transfer step to be limiting the reaction rate.

Kinetic isotope effect (KIE) experiments that compare activity in normal and deuterated water, in principle, may also allow one to differentiate between the chemical and PCET steps, since the former does not involve any proton donors. Wuttig et al.^3^ observed no discernable KIE and therefore postulated that CO$_{2}$ adsorption must be limiting the reaction rate. However, our calculations suggest that the conclusions from a KIE experiment are not necessarily unequivocal. As shown in Supplementary Table 5 (Supplementary Note 3), we predict different KIEs based on changes of the zero-point energy^30^ for the *CO$_{2}$ to *COOH step and the *COOH to *CO step, with the latter being essentially negligible. Therefore, it would be possible that the rate-limiting step corresponds to the proton–electron transfer to *COOH to form *CO, even when the KIE is negligible. In addition, the KIE effect can be convoluted with isotope effects related to mass transport, arising from differences in the diffusion constant of deuterated water or the acid dissociation constants, both of which would increase the local CO$_{2}$ concentration^31^.

### Multiscale modeling

**Fig. 3:**
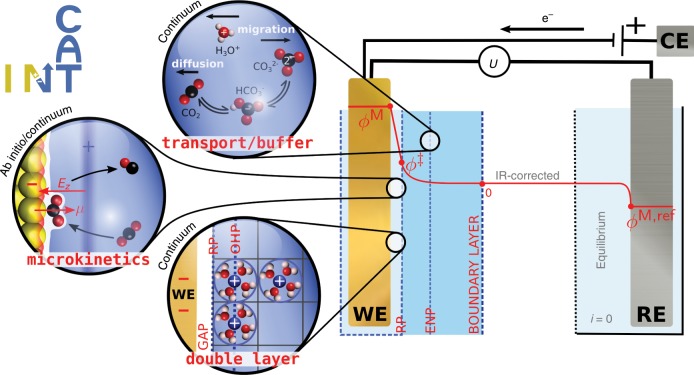
Schematic illustration of the multiscale approach to electrocatalytic CO$_{2}$ reduction. Reaction kinetics obtained from field-dependent DFT calculations are used to parameterize a microkinetic model. The model is coupled to a continuum mass transport model, which models the diffusion, migration, and reaction of species inside the boundary layer. This explicitly includes the double layer in which the electric field drives the reduction of CO$_{2}$. The double-layer model also includes the presence of a gap capacitance and cation repulsions at the outer Helmholtz plane (OHP), which are critical at the typically applied negative potentials. The thus-defined multiscale approach predicts the CO production rate as a function of the applied electrode potential $U={\phi }^{{\rm{M}}}-{\phi }^{{\rm{M}},{\rm{ref}}}+\Delta \mu^{\mathrm{M}}$, where Δ*μ*^M^ is the chemical potential difference between the electrodes and *U* has been corrected for the IR potential drop. The reference electrode is in electrochemical equilibrium, so that all fluxes *i* vanish. WE working electrode, RE reference electrode, CE counter electrode, RP reaction plane, ENP electroneutrality plane.

Figure 3 illustrates our integrated, multiscale approach to model electrocatalytic CO$_{2}$ reduction. At the ab initio scale, we determine surface-charge density $\sigma$-dependent reaction thermodynamics from density-functional theory (DFT) calculations using a continuum solvent and planar countercharge representation on various facets of Gold (cf. Supplementary Note 4, Supplementary Figs. 7–10, and Supplementary Table 6 for all results). The dependence of all thermodynamic states on the applied cell voltage $U$ is expressed via the computational hydrogen electrode approach (CHE)^32^. More details can be found in the “Methods” section and Supplementary Note 1. The resultant voltage- and surface-charge-dependent mean-field microkinetics are coupled to a Poisson–Nernst–Planck mass transport model integrating a continuum representation of the double-layer structure, the effect of cation–cation repulsion, and the associated finite ion size effects, diffusion, migration, and buffer equilibria within the boundary layer of the working electrode. In contrast to previous electrochemical mass transport models^8,12,16^, our scheme accounts for the electrostatic interaction of charged species with the charged electrode, which we show to be critical to rationalizing the CO$_{2}$R activity. We furthermore use Robin boundary conditions to relate the surface-charge density parameter in the DFT calculations to the applied cell voltage $\sigma (U)$. The self-consistent solution of steady-state kinetic rate and stationary mass transport equations has been implemented into our newly developed *Cat*alysis at the *I*nterfacial *N*ode to *T*ransport phenomena (CatINT) program package, which is described in detail in the Methods section of the paper and Supplementary Information (cf. in particular Supplementary Fig. 23 for a schematic representation).

### Computational evidence for the rate-limiting step

Recent experiments have indicated that CO_2_R on polycrystalline Gold happens predominantly at undercoordinated surface sites^19,34^. Following this, we considered a stepped (211) facet as the active site and refer the interested reader to Supplementary Note 5 for a more detailed discussion on the facet dependence. The (211) facet was chosen as a prototypical undercoordinated site based on previous theoretical data that suggested the *COOH binding energy to be relatively invariant with the step site model^19^. Recent studies on the CO$_{2}$ adsorption process showed that the transition state is structurally very close to the *CO$_{2}$ state^24^. Analogously, we found the *CO$_{2}$ to *COOH transition state to be close to *COOH by performing NEB–DFT calculations and extrapolating to a constant potential^33^ (cf. Supplementary Fig. 5). In the case of the *CO$_{2}$ state being less stable than the *COOH step, a concerted mechanism from CO$_{2({\rm{g}})}$ to *COOH could be possible. Using NEB calculations, we found this pathway, however, to be kinetically hindered (cf. Supplementary Fig. 6). These results suggests that the reaction likely happens via subsequent *CO_2_ adsorption and proton-coupled electron transfer to *COOH, with both states being reasonably well described by their thermodynamic stability. The *COOH to *CO step, in contrast, exhibits a potential-dependent kinetic barrier. We left this barrier as a variable parameter and discuss its influence on the obtained results in the section “Carbon dioxide adsorption”. The symmetry factor $\beta$ for this particular step was setchosen to be 0.5.

**Fig. 4:**
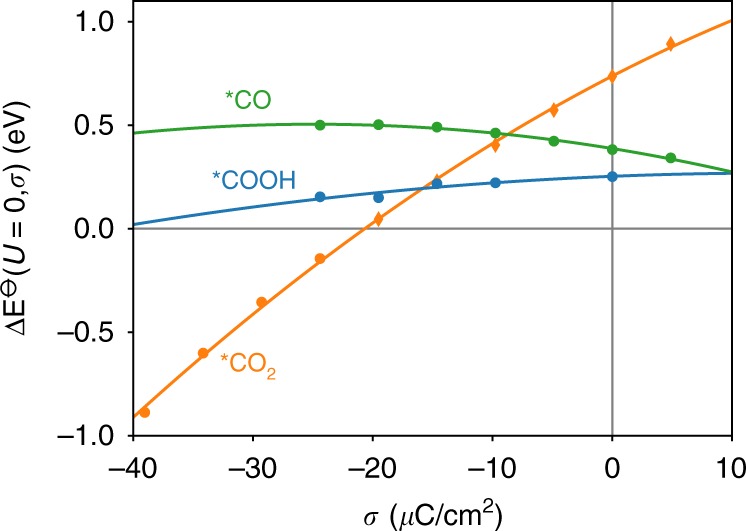
Surface-charge-dependent formation energies. The formation energies $$\Delta {E}^{-\ominus-}$$ are given for the key reaction intermediates *CO$$_{2}$$, *COOH, and *CO. Energies are referenced to CO$$_{2}$$ and H$$_{2}$$O in the gas phase, without corrections for the zero-point energy or finite temperature. The dependence on surface-charge density $$\sigma$$ at the (211) facet of Gold is shown without regard of the CHE–Frumkin potential-dependent term in Eq. (5). In the case of *CO$$_{2}$$, the formation energy for surface-charge densities $$> $$−20 µC/cm^2^ was obtained from fixed geometry, single-point calculations using a constrained *CO$$_{2}$$ geometry.

Figure 4 shows the dependence of the formation energy of all reaction intermediates on the surface-charge density. From this, we find *CO$_{2}$ to be strongly stabilized by increasing negative surface charge and the corresponding interfacial electric field, while *COOH and *CO are less substantially affected.

Using the surface-charge-dependent intermediate formation energies, we set up a mean-field microkinetic model for the elementary reaction steps given in Fig. 1. We adopted the pH = 6.8 experimental conditions that are presented in Fig. 2 and assumed water as a proton donor for the electrode reactions. Due to the neutral pH, we considered the presence of both proton- and hydroxide-driven buffer reactions, in contrast to previously published multiscale modeling of CO$_{2}$R^8,12^. HER was not included in the multiscale modeling approach for several reasons. First of all, previous theoretical studies found small binding energies of all reaction intermediates^26^, suggesting negligibly small coverages. Experiments based on ATR-SEIRAS spectroscopy^3,27^ have additionally not observed any higher coverages of species. These theoretical and experimental findings suggest that there is effectively no competition for active sites. HER could also be thought as changing the local pH and thus affecting CO production. We found, however, that the HER partial current density is lower than that of CO by orders of magnitude at pH = 6.8 in all but the highly mass transport-limited regions (<1.5 V vs. SHE) (cf. Supplementary Fig. 4). From this, we conclude that HER likely cannot change the pH enough to alter the CO current. This is supported by the fact that the CO evolution rate does not change when the bulk pH changes from 3.0 to 6.8, even though the HER rate is almost 100 times higher at pH 3.0 (cf. Fig. 2).

The mapping of the turnover frequency (TOF) obtained from the microkinetic model to a current density requires an estimate for the active site density. Recent experimental studies correlated the CO production activity with the density of grain boundaries on Gold surfaces^35^. The reported grain boundary density of 2.873 µm^−1^ for polycrystalline Gold corresponds to an active site density of ${\rho }_{{\rm{act}}}=9.6\cdot 1{0}^{-5}$ ${\rm{sites}}/{\mathrm{{\AA}} }^{2}$, considering (211)-like atomic distances. Using this value, we achieved a nearly quantitative prediction of the CO partial current density. We stress that a realistic value for the active site density is essential, since the absolute current density determines the mutual dependence of mass transport and kinetics, which depends sensitively on the absolute current. All other parameters for the multiscale model are documented in Supplementary Table 7 (Supplementary Methods).

**Fig. 5:**
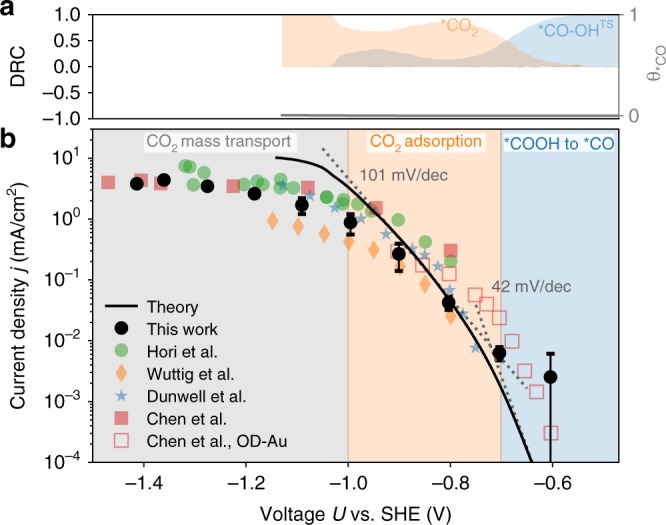
Multiscale CO$_{2}$ reduction simulation results compared with experiment. **a** Simulated degree of rate control (DRC), where a positive (negative) value indicates that the considered reaction intermediate needs to be stabilized (destabilized) in order to enhance the rate, and boundary values of 1 and −1 represent full rate control by the intermediate. *CO–OH refers to the *COOH to *CO transition state. The *CO coverage is shown as a gray line (plotted with respect to the right axis). **b** CO polarization curve, where the solid line shows the multiscale simulation result compared with experimental results from this work (0.1 M KHCO$_{3}$, pH = 6.8, black filled circles) and previous studies using 0.5 M KHCO$_{3}$ (pH = 7.2) of Dunwell et al.^6^, Wuttig et al.^3^, Hori et al.^36^, and Chen et al^37^. (filled colored symbols). Data have been generally obtained using polycrystalline Gold electrodes, despite the additionally shown data from ref. ^37^ on oxygen-derived nanoporous Gold electrodes (normalized using a roughness factor of 72). Tafel slopes from our model are indicated with the dotted line.

Figure 5 shows the CO partial current density curves as obtained from coupled micro-kinetic-transport modeling using CatINT. The curvature and slope of the curve show good agreement with the present experimental data and also with previous studies from Hori et al.^36^, Dunwell et al.^5^, Chen et al.^37^, and Wuttig et al.^3^. We performed a degree of rate control (DRC) analysis on our data, where the DRC is defined as the change in the CO production rate with the free energy of a particular state assuming constant activities of all species^38^:

1 $${{\rm{DRC}}}_{m}=\frac{{{d}}\ {\mathrm{log}}({r}_{{\rm{CO}}})}{-{{d}}({\tilde{\mu }}_{m}/{\mathrm{RT}})}.$$

${\tilde{\mu }}_{m}$ represents either of the electrochemical potentials of *CO$_{2}$, *COOH or *CO, or the *COOH to *CO transition state (“*CO-OH$^{{\rm{TS}}}$”) energy. From the DRC analysis, we found the *COOH to *CO transition state to exhibit the strongest influence on the CO production rate and thus to be rate limiting in the low-overpotential region. In the intermediate- and high-overpotential region, we found *CO$_{2}$ adsorption to be rate limiting instead (cf. Fig. 5a).

In the low-overpotential region, we found a small Tafel slope of 42 mV/dec, close to experimental measurements on oxygen-derived (OD) Gold (cf. Fig. 5b). OD-Gold electrodes, due to their high roughness factors, enable us to probe this low-current region. Since the *COOH to *CO step is limiting the reaction rate, the overpotential depends on the kinetic barrier of the *COOH to *CO step. At higher overpotentials ($<\! -\! 0.7$ V vs. SHE), the DRC suggests that *CO$_{2}$ adsorption controls the conversion to CO. This also suggests that *COOH to *CO cannot be limiting the reaction rate at these electrode potentials, because this would require a symmetry factor deviating significantly from the commonly considered 0.5^5^ in order to reproduce the same slope. Furthermore, the kinetic barrier for the *COOH to *CO step would need to be considerably larger than the chosen value of 0.6 eV at 0 V vs. SHE. For comparison, potential extrapolated NEB calculations using DFT found a kinetic *COOH to *CO barrier of only 0.66 eV on Pt(111)^39^. We thus conclude that *COOH to *CO is unlikely to limit the activity in higher-overpotential regions.

Finally, we also comment on the simulated *CO coverage. As shown in Fig. 5a, the *CO coverage is essentially zero over the whole potential range. Previous studies found a 20% *CO coverage at all potentials $<$−0.8 V vs. SHE using ATR-SEIRAS spectroscopy^3^, which was, however, later suggested by Dunwell et al.^6^ to arise from Pt impurities. The latter authors could not detect any *CO coverage under reducing conditions all the way down to −1.0 V vs. SHE, which is also in line with later studies that measured down to −0.9 V vs. SHE^27^. These results together with ours thus suggest that *CO coverage is indeed negligible under CO$_{2}$R conditions.

### Carbon dioxide adsorption

Considering CO$_{2}$ adsorption as the rate-limiting step, there are two major contributions to the apparent Tafel slope, ${A}_{{\rm{Tafel}}}$: the charging properties of the surface and the resulting stabilization of the *CO$_{2}$ state, as well as *CO$_{2}$ mass transport contributions. Since the coverages of all species are negligibly small, the Tafel slope can be expressed as follows (cf. Supplementary Note 6 for the derivation):

2 $$	{A}_{{\rm{Tafel}},1}\\ 	={\left|-{\mathrm{log}}_{10}(e)\frac{\Delta {a}_{\sigma,1}+2\Delta {b}_{\sigma,1}\sigma }{{\mathrm{RT}}}\underbrace{\frac{\partial \sigma }{\partial U}}_{{C}_{{\rm{dl}}}}+\frac{{{d}} \, {\mathrm{log}}({a}_{{{\rm{CO}}}_{2}}^{\ddagger })}{{\mathrm{d}}U}\right|}^{-1},$$

where ${a}_{{{\rm{CO}}}_{2}}^{\ddagger }$ is the activity of CO$_{2}$ at the reaction plane. In the first part, $\Delta {a}_{\sigma,1}$ and $\Delta {b}_{\sigma,1}$ depict the linear and quadratic surface-charge dependence of the *CO$_{2}$ formation energy (cf. Eq. (5) in the Methods section), where in this case $\Delta {b}_{\sigma,1}$ is relatively small. ${C}_{{\rm{dl}}}$ represents the double-layer capacitance, which is roughly constant with applied voltage (cf. Supplementary Fig. 12). Together, this causes the almost constant Tafel slope in the kinetic region of the polarization curve (cf. Fig. 5b). The reader is referred to Supplementary Note 6 for an analogous expression for the PCET steps.

As seen from Fig. 5b, this relationshipleads to a Tafel slope of 101 mV/dec for the CO$_{2}$ adsorption step, which agrees reasonably well with the experimentally reported range of values of 120–150 mV/dec in the intermediate-potential range. This good agreement with the experimental data can be seen as support of our reaction mechanism that considers field-driven CO$_{2}$ adsorption as the first reaction step, followed by two PCETs. Previous Tafel analysis assumed a full electron transfer to CO$_{2}$ and a symmetry factor of 0.5, which results in a Tafel slope of 120 mV/dec. In this work, we instead explicitly simulate the partial transfer of charge from *ab initio* that occurs with CO$_{2}$ adsorption, and the resultant dipole that interacts strongly with the interfacial field. The potential dependence of the interfacial field causes to the potential dependence of CO$_{2}$ adsorption.

We also performed experiments to evaluate the sensitivity of CO and H$_{2}$ production toward exchange of the electrolyte-containing cations (cf. Supplementary Note 8, Supplementary Fig. 22). From this, we found a strong cation effect on the CO production rate, while the HER rate was nearly unchanged. This is in line with our recent work^20^, which showed that smaller hydrated cations such as Cs$^{+}$ are highly concentrated in the Helmholtz layer, giving rise to an increased interfacial electric field. This makes cation exchange a way to probe the field sensitivity of the rate-limiting step, and in accordance with our results above, we find the CO production rate to be highly sensitive to cation identity, in contrast to the HER rate. Interestingly, we also found the transition between the rate-limiting steps to happen at a relatively higher overpotential for Cs$^{+}$, which could be due to a strong stabilization of *CO$_{2}$, making the *COOH to *CO step limiting until higher overpotentials.

### Mass transport of carbon dioxide

**Fig. 6:**
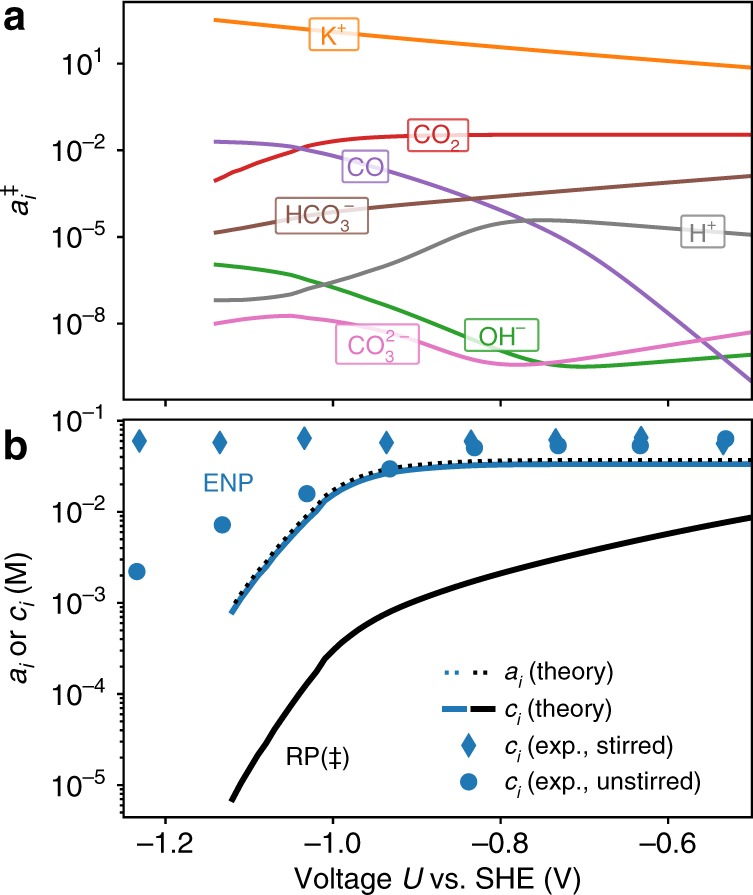
Voltage dependence of activities and concentrations. **a** Species activities at the reaction plane (RP, $\ddagger$) as a function of electrode potential $U$ as predicted by the coupled microkinetic mass transport modeling scheme using CatINT (bulk pH of 6.8). **b** CO$_{2}$ concentration and activity at the reaction plane (RP, $\ddagger$, black) and electroneutrality plane (ENP, 10 nm distance from RP, blue) compared with the ATR-SEIRAS spectroscopic results from ref. ^14^, all evaluated at a bulk pH of 7.3. The dots and diamonds denote nonstirred and stirred reaction conditions in the experiments, respectively.

The last term in Eq. (2) is defined by the mass transport of CO$_{2}$ to the electrode. Figure 6 shows the potential-dependent species activities at the reaction plane as obtained from our multiscale model (cf. also Supplementary Figs. 13, 14 for position- and potential-dependent concentrations, respectively). We found the CO$_{2}$ activity to be constant in the low-overpotential region and decreases due to CO$_{2}$ deficiency at the reaction plane at higher overpotentials. The decreased activity leads, according to Eq. (2), to an increase in the Tafel slope and the observed leveling off in the experimental and theoretical polarization curves (cf. Fig. 5b). By increasing the boundary-layer thickness, we found a decrease in the CO production current at high overpotentials, which confirms the CO$_{2}$ mass transport limitations (cf. Supplementary Fig. 15).

The double layer has two main effects on the mass transport. First, it leads to migration of positively charged species as K$^{+}$ or H$^{+}$ toward the negatively charged electrode, and repels anions (cf. Supplementary Fig. 16). Second, giventhe high applied overpotential and the positive PZC of Gold, the potassium cation concentration at the OHP is nearly fully saturated (cf. Supplementary Figs. 13, 14). We included the effect of ion crowding by introducing a lattice model with lattice sizes representing the size of each species within the so called Modified Poisson-Boltzmann approach (cf. Methods part). Within this approach, the included repulsive interactions limit the K$^{+}$ concentration and make it roughly constant over the considered voltage range. Besides K$^{+}$, other species concentrations are also affected by the repulsive interactions, changing, e.g., the local pH or the water dissociation equilibrium (cf. Supplementary Note 7 and Supplementary Figs. 13, 14, 17–19). In particular, the local CO$_{2}$ concentration at the reaction plane is reduced relative to the ENP as shown in Fig. 6b. In our model, we only included a finite species size for potassium cations which become highly concentrated at the electrode. However, we also tested the inclusion of a finite size of ${a}_{i}^{{\rm{cell}}}=4$ Å for all other species than potassium. Due to the much smaller concentration, we found this, however, to not have any effect on the activity coefficient (Eq. (9)) and also not on the CO production rate. The local depletion of species is thus only a function of the effective K$^{+}$ size used in the model. The catalytic conversion rate, however, depends only on the species’ activities, which are not affected by the repulsive interactions, due to the cancelation of the activity coefficient increase and the concentration decrease.

### Voltage-dependent species distribution

Recently, CO$_{2}$ concentration profiles were measured at a bulk pH of 7.3 using ATR-SEIRAS spectroscopy that effectively probes a region of around 5–10-nm distance to the electrode^14^. Figure 6b compares these experimental values with the calculated potential-dependent local CO$_{2}$ concentration at 10 nm from the reaction plane. In the following, we will refer to this distance as the electroneutrality plane (ENP, cf. Fig. 3), since the double layer extends to about this distance. Theoretical values at the ENP appear to be consistent with the experimental values under nonstirred experimental reaction conditions. The comparison also shows that although the CO$_{2}$ concentration at the reaction plane is affected by repulsive interactions, the CO$_{2}$ activity is not. The CO$_{2}$ activity is thus not subject to double-layer charging effects and is the same at both the reaction plane and ENP.

**Fig. 7:**
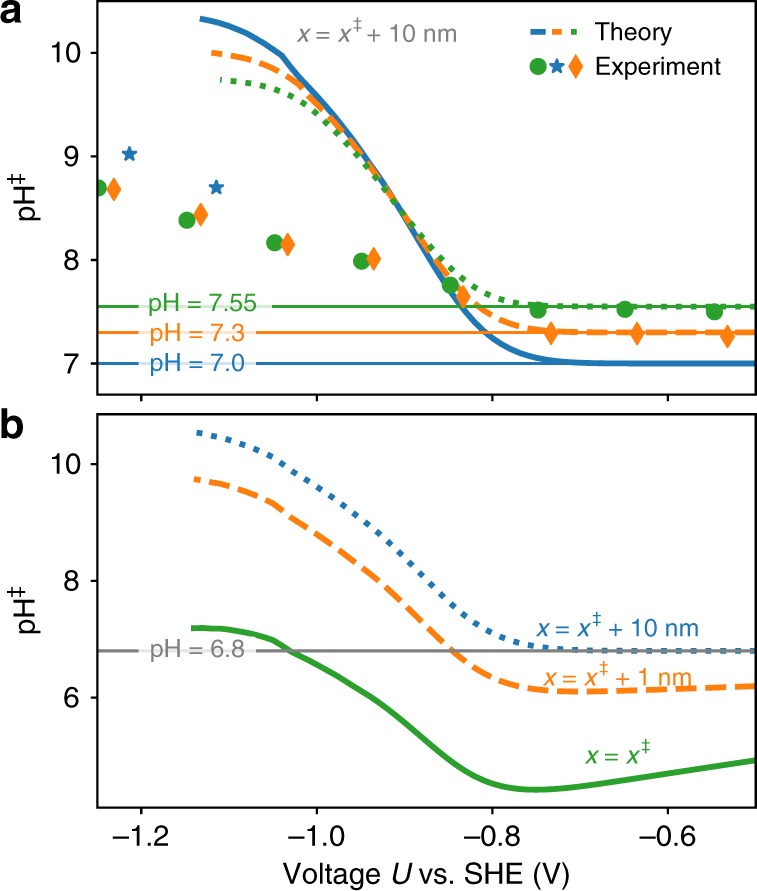
Voltage dependence of the local pH. **a** Local pH at the ENP (10 nm) as a function of electrode potential as predicted by the coupled microkinetic mass transport modeling scheme using CatINT (solid lines) and ATR-SEIRAS spectroscopy (symbols) of ref. ^14^ for three different bulk pH values. **b** Modeled local pH at three different distances from the electrode, the reaction plane (RP, $\ddagger$), 1 nm distance, and 10 nm distance (electroneutrality plane, ENP). The horizontal line indicates the bulk pH of the simulations and experiments.

Figure 7a shows the potential dependence of the local pH at the ENP compared with ATR-SEIRAS spectroscopy^14^. In agreement with the experimental data, we find the pH to behave bulk-like at lower overpotentials and increase due to the higher CO production rate and production of hydroxide anions at higher overpotentials^12,14^. The nonzero species fluxes at the electrode perturb the double layer and lead to concentration profiles deviating significantly from equilibrium Poisson–Boltzmann theory (cf. Supplementary Fig. 20). At high overpotentials, both theory and experiments predict inverse trends, with lower bulk pH values, resulting in a higher local pH at the ENP. We explain this by the increased bicarbonate buffer concentration in the higher bulk pH solutions, e.g., in the case of pH 7.55 (0.53 M KHCO$_{3}$) compared with pH 7.0 (0.15 M KHCO$_{3}$). This ensures a better buffering of the produced hydroxide anions at the electrode, keeping the pH closer to the bulk pH. We note that the overestimation of the pH in the high-overpotential region could be attributed to differences in the cell hydrodynamics, where our model predicts a smaller pH with increased boundary-layer thickness (inverse trend to what was observed before^12^). Also the HER, not considered in the theoretical model, could be sensitive to the bicarbonate concentration^29^, which would lead to local pH changes.

At the reaction plane, the generally negative surface charge at the electrode leads to an increase in the local concentrations of cations and a decrease in those of anions. The primary effect of this electrostatic interaction is the increased concentration of protons and the resultant stabilization of the *COOH state relative to the *CO$_{2}$ state by 120–240 meV (cf. Fig. 1). In consequence, *CO$_{2}$ adsorption becomes a more likely rate-limiting step in the production of CO. This result points to the importance of the double-layer structure on the activity, hitherto neglected in previous work.

### Bicarbonate reaction order

Since with CO$_{2}$ adsorption a pH-independent process is determining the reaction rate, the CO production rate depends only on the local concentration of CO$_{2}$ at the reaction plane. Recent studies further investigated the influence of bicarbonate on the CO production rate (bicarbonate reaction order) and obtained varying results^3,6^. The determination of the bicarbonate reaction order is complicated by the strong dependence on the local pH, diffusion, and migration effects as the coupling to the local CO$_{2}$ concentration. By varying the bicarbonate concentration, keeping the total ionic strength constant, and normalizing the current density using the local CO$_{2}$ concentration, Dunwell et al. found a 0.9 reaction order indicating the role of bicarbonate in transporting the reactive CO$_{2}$ species to the electrode^6^. Within our modeling approach, we test this finding directly by considering two separate CO$_{2}$ species, one reacting at the electrode, and the other participating in the buffer equilibria. By this, we found no change in the CO production rate compared with our above-discussed simulations, indicating that the buffer equilibrium does not produce reactive CO$_{2}$. This is also indicated by the buffer CO$_{2}$ activity being only slightly reduced at high overpotentials (cf. Supplementary Fig. 21).

## Discussion

Gold remains among the most active and selective CO$_{2}$R to CO electrocatalysts to date, and elucidating the mechanism for this process is therefore of critical importance to the development of improved CO$_{2}$R catalysts. In performing new CO$_{2}$R experiments at acidic pH values, we find the H$_{2}$ partial current density to substantially shift with pH on an absolute SHE potential scale, indicating a proton–electron transfer to be limiting the conversion rate. In contrast, we find that the CO production rate is insensitive to the bulk pH, which suggests that the first reaction step not involving the transfer of a proton is limiting the conversion rate.

In order to derive a more thorough understanding of the processes involved, we developed a new multiscale approach, coupling microkinetics with a detailed account of mass transport phenomena, such as diffusion, migration, and buffer reactions. In contrast to previous approaches, we explicitly integrated the structure of the electric double layer, which we found to be critical to determining the electrocatalytic activity.

By applying this model, we obtained excellent agreement with the major features of the experimental polarization curve and qualitative agreement with ATR-SEIRAS spectroscopic measurements of previous studies^14^. In detail, we rationalized the previously reported Tafel slope of around 40–60 mV/dec at very low overpotentials by a rate-limiting *COOH to *CO step and the Tafel slope of around 120–150 mV/dec at most of the relevant potential window by a rate-limiting CO$_{2}$ adsorption step and thereby resolved a long-standing controversy in the literature^3,5,6^. We found the measured Tafel slope for CO$_{2}$ adsorption to arise from the potential dependence of the surface-charge density, which stabilizes the dipolar *CO$_{2}$ state. The origin for the kinetic limitations is therefore not an electron transfer but the field-dependent stabilization of the *CO$_{2}$ state. At high overpotential, the CO current is limited by CO$_{2}$ diffusion limitations. Besides that, we also show that the negative charge of the electrode critically reduces the local pH at the reaction plane. Finally, our model shows that all reactive CO$_{2}$ diffuses to the electrode from the boundary layer and bicarbonate buffer does not significantly increase the amount of reactive CO$_{2}$ under stationary reaction conditions.

These new findings from experiments and multiscale modeling highlight the role of surface charging in electrocatalysis and open possibilities for improving catalyst-conversion efficiencies and product selectivities. In particular, non-PCET reaction steps that involve the transfer of partial electrons and the creation of a strong dipole moment as discussed here have been found to limit important electrochemical processes as the formation of C$_{2}$ products on Cu^17,18,20^. Our results suggest that such processes can be generally activated by improving the charging properties of the solid–liquid interface, by changing the potential of zero charge, e.g., via co-adsorbates or introducing impurities into the Gold electrode, by varying the Helmholtz gap capacitance, by changing the size or charge of the electrolyte-containing cations^20^, or by applying an external field via a field-effect transistor^40^. Further, the here-discovered sensitivity of the reactive pH on the charge of the electrode could be important to tune the product selectivity or activity of pH-sensitive reactions^41^. The new insights and identified descriptors therefore constitute an important piece toward the strategic optimization of electrochemical processes.

## Methods

### Experimental

The electrochemical testing setup used for CO$_{2}$R on Gold foil electrodes has been extensively described in the literature^42,43^. In brief, the custom-built electrochemical cell consists of two cell compartments partially filled with electrolyte and separated by an ion-conductive membrane. This electrochemical cell, characterized by a high ratio of electrode area (5.8 cm^2^) to electrolyte volume (10 mL), was used to conduct short-duration potentiostatic experiments using freshly cleaned Gold and platinum foils as the respective working and counter electrodes. Before each experiment, the Gold foil (thickness 0.1 mm, Alfa Aesar, 99.9975+% metal basis) was submerged in a 40 vol% aqueous nitric acid solution (Fisher Scientific, Certified ACS Plus) for at least 15 min to remove metal contaminants. The Gold foil was then rinsed with Millipore water and dried with a nitrogen flow before the assembly of the electrochemical cell. A similar pretreatment was carried out for the platinum foil used as a counter electrode. Three types of ion-exchange membranes were used in this work, depending on the pH and nature of the electrolyte tested. These were anion-exchange membranes for experiments near neutral pH (Selemion AMV; AGC), bipolar membranes (Fumasep FBM, FuMA-Tech GmbH, Germany) for experiments at pH 3.0, and proton-exchange membranes (Nafion 117, Fuel Cell Store Inc., USA) for experiments at pH 1.0. CO$_{2}$ gas (5.0, Praxair) was constantly bubbled at a rate of 20 sccm through both electrolyte compartments using a glass gas dispersion frit previously described^44^.

Two types of electrolytes were used for the CO$_{2}$R experiments, namely, buffered and unbuffered electrolytes. Buffered electrolytes such as bicarbonates and dihydrogen phosphates maintain a constant bulk pH in both cell compartments throughout the length of the experiment, independently of the ion membrane and pH used. On the other hand, electrochemical testing in unbuffered electrodes leads to the increase of pH in the working electrode, which is exacerbated at higher current densities. In order to alleviate the pH change with the progress of the experiment, a bipolar membrane was used to split water and generate protons and hydroxyl ions to respond to pH changes. However, it must be noted that bipolar membranes did not completely suppress pH variations for experiments carried out at pH 3.0 in the unbuffered KClO$_{4}$ electrolyte, and for these reasons, potentiostatic experiments were limited to only 4 min in duration. These short experiments are enough to obtain a complete product quantification, while limiting pH variations to below 0.5 units of pH even in high-current experiments. Experiments at pH 1.0 used a cation-exchange membrane and did not result in any measurable pH variations. High-purity KHCO$_{3}$ (Sigma Aldrich, 99.99% trace metal basis), KClO$_{4}$ (Sigma Aldrich, 99.99% trace metal basis), KH$_{2}$PO$_{4}$ (Sigma Aldrich, purity $\ge$99.0%), perchloric acid (70% HClO$_{4}$, Suprapur, Merck), CsOH (Sigma Aldrich, 99.95% trace metal basis), and NaHCO$_{3}$ (Sigma Aldrich, $\ge$99.7% ACS reagent) were obtained from commercial sources and used without any further purification to prepare the electrolyte solutions. All aqueous electrolytes were prepared using high-purity Millipore water. Gas products were detected using an online gas chromatography. The boundary-layer thickness in this electrochemical cell has been determined to be 80 µm, using the diffusion-limited current for ferricyanide reduction^45^.

### Voltage- and surface-charge-dependent kinetics

Figure 1 illustrates the CO production rate expressions resulting from the different steps being limiting. The expressions are written as a function of the applied electrode potential $U$:

3 $$U=\Delta {\phi }^{{\rm{M}}}+\Delta {\mu }^{{\rm{M}}} .$$

Here, $\Delta {\phi }^{{\rm{M}}}={\phi }^{{\rm{M}}}-{\phi }^{{\rm{M}},{\rm{ref}}}$ refers to the working electrode electrostatic potential relative to the reference electrode (here the standard hydrogen electrode − SHE) and $\Delta {\mu }^{{\rm{M}}}$ is the chemical potential difference of both electrodes. The driving force for any coupled proton–electron transfer referenced to SHE is given by

4 $${U}_{{\rm{F}}}=U+(2.3\,{\mathrm{RT}}\,{{\rm{pH}}}^{\ddagger }/F-{\phi }^{\ddagger }) .$$

Here, ${{\rm{pH}}}^{\ddagger }$ refers to the pH, and ${\phi }^{\ddagger }$ to the electrostatic potential at the reaction plane (RP, $\ddagger$). The additional terms that correct $U$ are generally referred to as Frumkin^46^ corrections. They arise from the potential drop in the diffuse layer of the working electrode. We note that the IR potential drop is usually corrected out in experiments and thus ignored here. All potentials are shown in Fig. 3 and are given relative to the bulk electrolyte.

Considering this corrected driving force, we set up a thermodynamic model to describe electrochemical reaction steps based on the Frumkin-corrected^46^ computational hydrogen electrode (CHE)^32^. For a transfer of $n$ proton–electron pairs, the free reaction energy change from the initial to the final state becomes

5 $$\Delta {G}_{m}(U)=	 \; \Delta {G}_{m}(U=0,\sigma =0)+\underbrace{nF\left(U-{\phi }^{\ddagger }\right)}_{{{\rm{full}}}\; {\rm{CT}}/{\rm{CHE}}-{\rm{Frumkin}}}\\ 	+\underbrace{\Delta {a}_{\sigma,m}\sigma (U)+\Delta {b}_{\sigma,m}{\sigma }^{2}(U)}_{{{\rm{partial}}}\; {\rm{CT}}/{\rm{field}}-{\rm{dependence}}},$$

where $m$ refers to the index of the reaction step, $F$ the Faraday constant, and $\sigma$ the surface-charge density. $a/{b}_{\sigma,m}$ are parameters representing the surface-charge dependence of a particular state and $\Delta {G}_{m}^{\circ }(U=0,\sigma =0)$ is the reaction energy of a hypothetical reference state at which the electrons are at the same electrochemical potential as the reference electrode ($U=0$) and no surface charge is present ($\sigma=0$). This state is hypothetical, because in reality the surface charging relation $\sigma(U)$ will prevent both $U$ and $\sigma$ to be zero at the same time, if not the PZC is also zero. The CHE model considers the potential dependence that evolves from the transfer of integer numbers of electrons and protons between the counter and working electrode via a coupled process^47^. This expression is extended by the sensitivity of the intermediate reaction states toward nonvanishing surface-charge density at a particular applied electrode potential (cf. Supplementary Information for a detailed explanation and rationalization of our approach).

Implicit solvation schemes have become extremely popular, in particular in electrochemistry^20,48–53^, not only due to their efficiency in treating solvation effects, but also the ability to incorporate simplified representations of countercharges. Here, we obtain the dependence of the reaction states on $\sigma$ by fitting $\sigma$-dependent implicit solvent DFT calculations using a planar countercharge representation via the Environ module^54^ of the DFT program package QUANTUM ESPRESSO^55^. The dependence on the surface charge is considered up to second order. Potential-dependent creation of surface charge ${\sigma(U)}$ can be obtained by integrating experimental double-layer capacitance measurements as a function of electrode potential^56^. Alternatively, continuum theories can be utilized to provide an estimate of the double-layer capacitance contribution, such as that described in the next section. We recently showed that surface-charge-dependent reaction energetics effectively captures the physics at the electrified solid–liquid interface, and enables us to elucidate the effect of cations on the electrochemical CO$_{2}$ reduction rate^20^.

We apply the Butler–Volmer approximation and consider a linear dependence on the potential (cf. Supplementary Information)

6 $${G}_{{\rm{a}},{\rm{m}}}^{-\ominus-}(U)={G}_{{\rm{a}}}^{\circ }(U(\sigma =0))+\underbrace{\beta FU}_{{{\rm{partial}}}\; {\rm{CT}}\,({\rm{Butler}}-{\rm{Volmer}}-{\rm{Frumkin}}+{\rm{field}}-{\rm{dependence}})}.$$

where $\beta$ is the symmetry factor. $\beta$ depends on the amount of charge transferred to the transition state, the charge symmetry of the transition state, and the field dependence of the activation energy. Higher-order terms are ignored for simplicity.

### Mass transport and double-layer charging

We consider mass transport of CO$_{2}$, CO, protons, hydroxide anions, as well as the buffer components K$^{+}$, HCO$_{3}^{-}$, and CO$_{3}^{2-}$ within a stationary, 80-µm-thick boundary layer (cf. Fig. 3). The transport of species in the electrolyte is modeled by the Nernst–Planck (NP) equation, in a generalized stationary form as

7 $$\nabla \cdot {i}_{i}=	 \, {R}_{i}\\ {i}_{i}= 	\,-\frac{{D}_{i}}{{{RT}}}{c}_{i}\nabla {\tilde{\mu }}_{i}\\ {\tilde{\mu }}_{i}=	 \, {\mu }_{i}^{\circ }+{{RT}}\ {\mathrm{ln}}(\underbrace{{\gamma }_{i}{c}_{i}}_{{a}_{i}})+{z}_{i}F\phi,$$

with the species concentrations ${c}_{i}$, activities ${a}_{i}={\gamma }_{i}{c}_{i}$, activity coefficients ${\gamma }_{i}$, fluxes ${i}_{i}$, diffusion constants ${D}_{i}$, the ideal gas constant $R$, the electrochemical potentials ${\tilde{\mu }}_{i}$, the species concentrations in the bulk solvent (outside the boundary layer) ${c}_{i}^{\circ }$, the charges ${z}_{i}$, and the electrostatic potential $\phi$. ${R}_{i}$ in Eq. (7) denotes a different source for the species $i$, as for example, buffer reactions that we include as kinetic rate expressions in the line of ref. ^12^ (cf. also Supplementary Information for all reaction equations). Coupled with the Poisson equation

8 $${\varepsilon }_{{\rm{b}}}\frac{{{\mathrm{d}}}^{2}\phi }{{\mathrm{d}}{x}^{2}}=-F\sum _{i}^{N}{z}_{i}{c}_{i}[\phi ]=-\rho [\phi] ,$$

with the bulk dielectric permittivity ${\varepsilon }_{{\rm{b}}}$ and charge density $\rho$, we have the well-known Poisson–Nernst–Planck (PNP) equations. Under typical experimental reaction conditions, a constant flow of reactants is provided, so we therefore assume constant concentrations at the end of the boundary layer (cf. Fig. 3).

We note that in this work, in contrast to previous models, we consider explicitly the structure of the double layer. The resultant equation system is challenging, since it is highly nonlinear and includes variations within two vastly different length scales. For this reason, the electric double-layer structure is mostly neglected in previous models through assuming global electroneutrality^8,16,57,58^. As discussed in the main text, however, even in the presence of a concentrated supporting electrolyte, the double layer does play a major role in both driving field-dependent electrochemical processes and in controlling the concentrations of charged species such as bicarbonate or protons at the reaction plane.

We also note that the activity coefficients ${\gamma }_{i}$ are generally not 1 at the reaction plane. Considering the positive PZC of Gold at 0.16 V vs. SHE^59^, the surface is expected to be highly negatively charged under CO$_{2}$R conditions (−0.9 V vs. SHE), which leads to high supporting electrolyte cation concentrations. As a result, highly concentrated cations induce repulsive interactions with all species, which reduces their respective local concentrations. In this work, we take the approach of the size-modified Poisson–Boltzmann model (MPB) (cf. Fig. 3a)^60^. Essentially, the MPB model introduces a statistical lattice model in which each cell is only allowed to be occupied by a single species. The model can be generalized by assigning different lattice cell sizes ${a}_{i}^{{\rm{cell}}}$ to each species, which leads to the activity coefficients^61^

9 $${\gamma }_{i}=\frac{1}{1-{N}_{{\rm{A}}}\sum _{i}^{N}{c}_{i}{{a}_{i}^{{\rm{cell}}}}^{3}} .$$

The activity coefficients of all species are therefore reduced if the size of a particular species is increased. For species with small concentrations, the contribution to the activity coefficient is small. For this reason, we considered a nonzero-size ${a}_{i}^{{\rm{cell}}}$ only for the highly concentrated and also due to its hydration shell large potassium cations, while the size of all other much less concentrated species was set to zero. The validity of this assumption is further discussed in the “Results” section. The K$^{+}$ cation diameter was chosen to be ${a}_{{{\rm{K}}}^{+}}^{{\rm{cell}}}=8.2$ Å based on the K$^{+}$–Ag(111) distance obtained from X-ray diffraction studies^62^. The whole set of Eqs. (7)–(9) defines the generalized modified PNP (GMPNP) mass transport model used in this study.

The one-dimensional GMPNP equation is solved using the boundary conditions in Table 1. Microkinetics and mass transport are coupled via a flux boundary condition at the reaction plane. The solution $\phi$ of the Poisson equation is Eq. (8). The potential in the bulk solvent ${\phi }^{{\rm{b}}}$ is set to zero by applying Dirichlet boundary condition The potential at the reaction plane ${\phi }^{\ddagger }$ is coupled to its gradient (proportional to the surface-charge density) through a Robin boundary condition at the reaction plane (cf. ref. ^63^ and Supplementary Information)

10 $$\sigma =-{\varepsilon }_{{\rm{b}}}{\left.\frac{{\mathrm{d}}\phi }{{\mathrm{d}}x}\right|}_{{x}^{\ddagger }}={C}_{{\rm{gap}}}\left[U-{U}^{{\rm{PZC}}}-{\phi }^{\ddagger }\right],$$

where ${U}^{{\rm{PZC}}}$ is the PZC of the working electrode relative to the reference electrode. Note that the surface charge does not depend on the potential reference electrode but only on the potential drop between the reaction plane and metal relative to the PZC. The gap capacitance ${C}_{{\rm{gap}}}$ arises from Pauli repulsion at the surface–water interface, which constitutes a significant part of the Helmholtz capacitance^64,65^. The constants defining the additional potential drop over the Helmholtz gap are the measurable cell potential $U$ and the PZC, both given relative to an absolute reference potential (here SHE). We consider the value of ${C}_{{\rm{gap}}}=20$ µC/cm^2^ for the gap capacitance according to the experimentally measured double-layer capacitance^66–68^ over almost the whole considered potential range (cf. Supplementary Fig. 12). All other parameters are explicitly given and explained in Supplementary Information. We define $x={x}^{\ddagger }$ as the reaction plane similar to previous theoretical studies, which located it at the outer Helmholtz plane (OHP)^69–71^. Importantly, both choices lead to reactants being embedded into the double layer and therefore their concentrations are sensitive to electrode-charging effects.

**Table 1:** Boundary conditions used for the solution of the GMPNP equations.

Equation	Boundary	Boundary condition
NP	$x={x}^{\ddagger }(=0)$	${j}_{i}^{{\rm{GMPNP}}}={j}_{i}^{{\rm{MK}}}$
NP	$x={x}_{{\rm{bl}}}$	${c}_{i}={c}_{i}^{0}$
Poisson	$x={x}^{\ddagger }(=0)$	Robin
Poisson	$x={x}_{{\rm{bl}}}$	$\phi =0$

*GMPNP* generalized modified Poisson–Nernst–Planck, *bl* boundary layer

${j}_{i}^{{\rm{GMPNP}}}$ and ${j}_{i}^{{\rm{MK}}}$ denote the species fluxes in the GMPNP mass transport model and microkinetic model, respectively

## Supplementary information

Supplementary Information

## Data Availability

All raw data forthe surface charge density-dependent DFT calculations of all reaction intermediates as well as the experimental CO$_{2}$ reduction experiments are provided in Supplementary Information. Other data are available on request from the authors.

## Code availability

CatINT is available free of charge at https://github.com/sringe/CatINT under the GNU GENERAL PUBLIC LICENSE. A program package documentation is provided at https://catint.readthedocs.io/en/latest.
