# Supplementary material for: Double layer charging driven carbon dioxide adsorption limits the rate of electrochemical carbon dioxide reduction on Gold
Source: Nat Commun. 2020 Jan 7;11:33. doi: 10.1038/s41467-019-13777-z (PMC6946669; doi:10.1038/s41467-019-13777-z)
Supplement: Supplementary file 1 — Supplementary Information [file 41467_2019_13777_MOESM1_ESM.pdf]

Supplementary Information

Double layer charging driven carbon dioxide adsorption limits the rate of electrochemical carbon dioxide reduction on Gold

Ringe *et al.*

Supplementary Note 1: Electrochemical model formulation

Reaction thermodynamics. We consider the free energy change associated with a particular reaction step

$$\mathrm{A} + n\,(\mathrm{H}^+ + \mathrm{e}^-) \rightleftharpoons \mathrm{B} \quad , \qquad \text{(Supplementary Eq. 1)}$$

where A and B refer to initial and final states, and n protons and electrons are transferred. The free energy change for this reaction step m is given by:

$$\Delta G_m = \sum_i \nu_i \tilde{\mu}_i = \tilde{\mu}_B - \tilde{\mu}_A - n(\tilde{\mu}_{\mathrm{H}^+} + \tilde{\mu}_{\mathrm{e}^-}) \quad , \qquad \text{(Supplementary Eq. 2)}$$

where the sum goes over all reactants and products and ν_i refers to the reaction equivalent. $\tilde{\mu}$ represents electrochemical potentials defined as

$$\tilde{\mu}_i = \mu_i + z_i F \phi^{\ddagger} \quad , \qquad \text{(Supplementary Eq. 3)}$$

where z_i is the charge of the reactant or product and $\phi^{\ddagger}$ is the electrostatic potential at the reaction plane relative to our reference, the bulk electrolyte. The proton and electron's electrochemical potentials can be described by the Computational Hydrogen Electrode (CHE),[1] which assumes electrochemical equilibrium at the reference Standard Hydrogen Electrode (SHE):

$$\tilde{\mu}^{\circ}_{\mathrm{H}^+} + \tilde{\mu}^{\circ}_{\mathrm{e}^-} = 1/2\mu^{\circ}_{\mathrm{H}_2} \quad , \qquad \text{(Supplementary Eq. 4)}$$

where μ denotes a chemical potential. The reactive proton, hydroxide and electron electrochemical potentials can be expressed as:

$$\tilde{\mu}_{\mathrm{H}^+} + \tilde{\mu}_{\mathrm{e}^-} = 1/2\mu^{\circ}_{\mathrm{H}_2} \underbrace{-2.3RT\mathrm{pH}^{\ddagger} + F\phi^{\ddagger}}_{\tilde{\mu}^{\mathrm{ex}}_{\mathrm{H}^+}} \underbrace{-FU}_{\tilde{\mu}^{\mathrm{ex}}_{\mathrm{e}^-}} \qquad \text{(Supplementary Eq. 5a)}$$

$$\tilde{\mu}_{\mathrm{OH}^-} = \mu_{\mathrm{H_2O}} - \tilde{\mu}_{\mathrm{H}^+} \quad , \qquad \text{(Supplementary Eq. 5b)}$$

where "ex" denotes the deviations of the electrochemical potentials from the reference state "∘". For example, the local pH at the reaction plane is different from the reference (pH = 0 at the SHE), requiring to shift the chemical potentials. Further, the protons are at a different electrostatic potential at the reaction plane than the CHE equilibrated protons ($\phi = 0$), due to the presence of the diffuse layer. Finally, electrons have a different electrochemical potential than the reference electrode, due to a different electrostatic potential $\Delta\phi^{\mathrm{M}}$ and a different chemical potential. The sum of these, is what is measured as a voltage difference between the reference and working electrode and referred to as $U = \tilde{\mu}^{\mathrm{ex}}_{\mathrm{e}^-}/F = \Delta\phi^{\mathrm{M}} + \Delta\mu_{\mathrm{e}^-}/F$. We name this part of the potential dependence *faradaic*, because it describes an electron transfer from counter electrode to working electrode. For the second equality, we assumed water self-dissociation equilibrium in the bulk.

For the here considered CO_2R process, all reaction intermediates are charge neutral. The electrochemical potentials of A and B thus are equivalent to the chemical ones:

$$\tilde{\mu}_{\mathrm{A/B}} = \mu_{\mathrm{A/B}} = \mu^{\circ}_{\mathrm{A/B}} + RT \ln a^{\ddagger}_{\mathrm{A/B}} \quad , \qquad \text{(Supplementary Eq. 6)}$$

where $a^{\ddagger}_{\mathrm{A.B}}$ is the activity of A at the reaction plane and $\mu^{\circ}_{\mathrm{A/B}}$ the chemical potential at standard conditions as can be derived from DFT calculations. The chemical potentials depend on the applied electrode potential, because the adsorbed states are sensitive to the potential-dependent electric double layer field. This effect is considered by a 2nd order Taylor expansion of the reaction free energy as a function of the surface charge density:

$$\Delta G_m(U=0,\sigma) = \Delta G_m(U=0,\sigma=0) + \Delta a_{\sigma,m}\sigma + \Delta b_{\sigma,m}\sigma^2 \quad , \qquad \text{(Supplementary Eq. 7)}$$

where $a/b_{\sigma,m}$ represent the dependence of the a state m on the surface charge density, and the Δ refers to the change from A to B. The parameters a_σ and b_σ are related to the theoretical PZC and capacitance of the electrochemical cell used to evaluate ΔG°_m.[2] $\Delta G_m(\sigma=0, U=0)$ depicts the free energy change at an a hypothetically applied potential vs. SHE of 0 V and a surface charge of 0. One of the two parameters can be eliminated, by defining a surface charging relation $\sigma(U)$. This can be e.g. obtained from experimental data or more accurate theoretical models.

The decoupling of the surface charging from the charge-dependent kinetics is one of the advantages in performing constant charge calculations in favor of the more frequently used constant potential simulations. The decoupling, makes Supplementary Eq. 7 relatively independent of the electrolyte,[3] and even added explicit solvent[4] which makes it a highly consistent way of defining the field-dependence.

In this work, we apply the surface charging function that is provided by the Robin boundary condition $\sigma(U) = C_{\rm gap}(U - U^{\rm PZC} - \phi^{\ddagger}) = \sigma(\Delta\phi^{\rm M}) = C_{\rm gap}(\Delta\phi^{\rm M} - \Delta\phi^{\rm M,PZC} - \phi^{\ddagger})$. However, for generality in the derivation, we in the following proceed without specifying the actual surface charging relation. By adding the potential dependence from the CHE model and combining all derived electrochemical potentials, we then arrive at an expression for the total free energy change of the reaction step:

$$\begin{aligned}\Delta G_m(U) &= \Delta G^{\circ}_m + \Delta a_{\sigma,m}\sigma(U) + \Delta b_{\sigma,m}\sigma^2(U) + RT\ln\left(\frac{a^{\ddagger}_{\rm B}}{a^{\ddagger}_{\rm A}}\right) + n\underbrace{\left(2.3RT{\rm pH}^{\ddagger} + F(U-\phi^{\ddagger})\right)}_{FU_{\rm F}} \\ &= \Delta G^{\ominus}_m(U) + RT\ln\left(\frac{a^{\ddagger}_{\rm B}}{a^{\ddagger}_{\rm A}}\right) + n2.3RT{\rm pH}^{\ddagger} \\ &= \Delta G_m(U=0,\sigma=0) + \Delta a_{\sigma,m}\sigma(U) + \Delta b_{\sigma,m}\sigma^2(U) + nF(U-\phi^{\ddagger}) \quad , \qquad \text{(Supplementary Eq. 8)}\end{aligned}$$

where we introduced the Frumkin-corrected electrochemical driving force (potential) $U_{\rm F}$. In line with the definitions before, ΔG°_m represents the free energy change at a hypothetical reference state, which in our case is the CHE with species activities of 1 and an applied voltage of $U = 0$ as well as $\sigma = 0$. The state is hypothetical, because if the PZC does not equal zero, U and σ can never be zero at the same time. ΔG°_m is the energy obtained from DFT at $\sigma = 0$ for the adsorbate state minus the bare slab and molecular gas reference states after addition of zero point energy and finite temperature corrections. $\Delta G^{\ominus}_m$ is the potential-dependent free energy change (incorporating also field effects) for standard activities of 1 for all species. The last line now separates the *faradaic* (defined by transfer of electrons from the counter electrode, which electrostatic potential can be tuned) and *capacitive* (defined by surface charging which stabilizes the adsorbate state relative to the bare slab) components of the reaction free energy. The former is relevant for any electron transfer reaction, while the latter should be considered for all reaction steps, but is in particular relevant for the adsorption of CO_2 which gives rise to large values for $\Delta a_{\sigma,m}$.

Reaction kinetics. The Brønsted-Evans-Polanyi (BEP) scaling relations relate the activation energy of a process $G^{\ominus}_{\rm a,m}$ to the reaction free energie $\Delta G^{\ominus}_m$.[5] We use the same approach and additionally include the charging contribution in the scaling:

$$\begin{aligned}G^{\ominus}_{\rm a,m}(U) &= RT\ln\left(\gamma^{\ddagger}\right) + \beta'\cdot\Delta G^{\ominus}_m(U) \qquad \text{(Supplementary Eq. 9)} \\ &= \underbrace{RT\ln\left(\gamma^{\ddagger}\right) + \beta'\cdot\Delta G^{\circ}_m}_{G^{\circ}_{\rm a,m}} + \beta'\cdot\left(\Delta a_{\sigma,m}\sigma(U) + \Delta b_{\sigma,m}\sigma^2(U) + nF(U-\phi^{\ddagger})\right) \\ &\approx G^{\circ}_{\rm a,m} + \beta FU \qquad \text{(Supplementary Eq. 10)}\end{aligned}$$

where $\gamma^{\ddagger}$ is the activity coefficient of the transition state defined as in ref.[5]. Here, we considered the linear dependence of surface charge density on $\Delta\phi$ in the applied Robin boundary condition. Further neglecting the second order term, results in a simple linear dependence of the activation energy on potential as in the original Butler-Volmer kinetics. The effective charge transfer coefficient β includes thus here both the effects of field stabilization and electron transfer symmetry of the reaction.

Kinetic rate expressions for carbon dioxide reduction. Under acidic conditions (proton as proton donor), the CO production rate can be expressed depending on the rate-limiting step as (indices as in Figure 2 of the main

manuscript):

$$
\begin{aligned}
&[1] \quad r_1 \propto a^{\ddagger}_{CO_2} \theta_* \exp\left(-\frac{G^{\ominus}_{a,1}(U)}{RT}\right) \\
&[2] \quad r_2 \propto a^{\ddagger}_{CO_2} \theta_* Q_1 \exp\left(-\frac{G^{\ominus}_{a,2}(U)}{RT} - 2.3\text{pH}^{\ddagger}\right) \\
&[3] \quad r_3 \propto a^{\ddagger}_{CO_2} \theta_* Q_1 Q_2 \exp\left(-\frac{G^{\ominus}_{a,3}(U)}{RT} - 2.3\text{pH}^{\ddagger}\right) \\
&[4] \quad r_4 \propto a^{\ddagger}_{CO_2} \theta_* Q_1 Q_2 Q_3 \exp\left(-\frac{G^{\ominus}_{a,4}(U)}{RT}\right) \quad ,
\end{aligned}
\qquad \text{(Supplementary Eq. 11)}
$$

where θ_* is the coverage of empty active sites and

$$
\begin{aligned}
Q_1 &= \exp\left(-\frac{\Delta G^{\ominus}_1(U)}{RT}\right) \\
Q_2 &= \exp\left(-\frac{\Delta G^{\ominus}_2(U)}{RT} - 2.3\text{pH}^{\ddagger}\right) \\
Q_3 &= \exp\left(-\frac{\Delta G^{\ominus}_3(U)}{RT} - 2.3\text{pH}^{\ddagger}\right)
\end{aligned}
\qquad \text{(Supplementary Eq. 12)}
$$

Note, that in this paper, we assume that the activation energy equals the reaction energy for the $^*CO_2$ to *COOH and *COOH to *CO reaction steps. Under alkaline conditions (water as proton donor), the rate becomes

$$
\begin{aligned}
&[1] \quad r_1 \propto a^{\ddagger}_{CO_2} \theta_* \exp\left(-\frac{G^{\ominus}_{a,1}(U)}{RT}\right) \\
&[2] \quad r_2 \propto a^{\ddagger}_{CO_2} \theta_* Q_1 \exp\left(-\frac{G^{\ominus}_{a,2}(U)}{RT}\right) \\
&[3] \quad r_3 \propto a^{\ddagger}_{CO_2} \theta_* Q_1 Q_2 \exp\left(-\frac{G^{\ominus}_{a,3}(U)}{RT}\right) \\
&[4] \quad r_4 \propto a^{\ddagger}_{CO_2} \theta_* Q_1 Q_2 Q_3 \exp\left(-\frac{G^{\ominus}_{a,4}(U)}{RT}\right) \quad .
\end{aligned}
\qquad \text{(Supplementary Eq. 13)}
$$

Supplementary Note 2: Experimental faradaic efficiencies

The following tables summarize the faradaic efficiencies measured for the CO_2 reduction experiments on polycrystalline Gold at different bulk pH values.

Supplementary Table 1: Faradaic efficiencies for pH = 1 CO_2R reaction conditions.

**Voltage** U **vs. SHE (V)**	**Current Efficiency (%)** H_2
-0.10283	0.97585
-0.20226	0.93138
-0.30078	0.96426
-0.39285	0.96335
-0.43639	0.96255
-0.47856	0.98217
-0.51809	0.9795

Supplementary Table 2: Faradaic efficiencies for buffered pH = 3 CO_2R reaction conditions.

**Voltage** U **vs. SHE (V)**	**Current Efficiency (%)** H_2	**Current Efficiency (%)** (%) **CO**
-0.59664 (±8.63E-04)	0.8331 (±0.11795)	0.00815 (±0.00332)
-0.79125 (±0.00566)	0.7723 (±0.02489)	0.1034 (±0.0577)
-0.95313 (±0.00407)	0.6373 (±0.1847)	0.0663 (±0.00721)
-1.13428 (±0.00844)	0.6156 (±0.06534)	0.35115 (±0.03316)
-1.304 (±0.00672)	0.42945 (±0.05084)	0.47305 (±0.03062)
-1.46175 (±0.00919)	0.63065 (±0.0647)	0.3139 (±0.0478)

Supplementary Table 3: Faradaic efficiencies for unbuffered pH = 3 CO_2R reaction conditions.

**Voltage** U **vs. SHE (V)**	**Current Efficiency (%)** H_2	**Current Efficiency (%)** (%) **CO**
-1.4574 (±3.75E-03)	0.40094 (±0.22991)	0.52524 (±0.02854)
-1.27846 (±0.00253)	0.11045 (±0.0586)	0.8284 (±0.16164)
-1.09061 (±0.0017)	0.06229 (±0.03654)	0.80995 (±0.07727)
-0.89809 (±0.00177)	0.30935 (±0.22386)	0.34942 (±0.1085)
-0.69823 (±0.0014)	0.72035 (±0.04398)	0.01568 (±0.0117)
-0.5016 (±0.00108)	0.76158 (±0.01541)	0.00276 (±0.00251)

Supplementary Table 4: Faradaic efficiencies for pH = 6.8 CO_2R reaction conditions.

**Voltage U vs. SHE (V)**	**Current Efficiency (%) H_2**	**Current Efficiency (%)** (%) **CO**
-0.60387 (±5.19E-04)	0.53989 (±0.28368)	0.15125 (±0.18558)
-0.70378 (±5.01E-04)	0.42158 (±0.27287)	0.16721 (±0.05922)
-0.80326 (±5.77E-05)	0.40696 (±0.16371)	0.35121 (±0.05684)
-0.90076 (±8.14E-04)	0.2893 (±0.19807)	0.65324 (±0.17392)
-0.9953 (±0.00266)	0.13927 (±0.11389)	0.79674 (±0.11144)
-1.09053 (±0.00458)	0.11864 (±0.09792)	0.97769 (±0.03238)
-1.18331 (±0.00568)	0.10104 (±0.06214)	1.0149 (±0.15698)
-1.27539 (±0.00579)	0.11899 (±0.03888)	0.96845 (±0.19397)
-1.36065 (±0.01183)	0.21502 (±0.03079)	0.87236 (±0.19001)
-1.4137 (±0.0178)	0.72383 (±0.20609)	0.35873 (±0.13756)

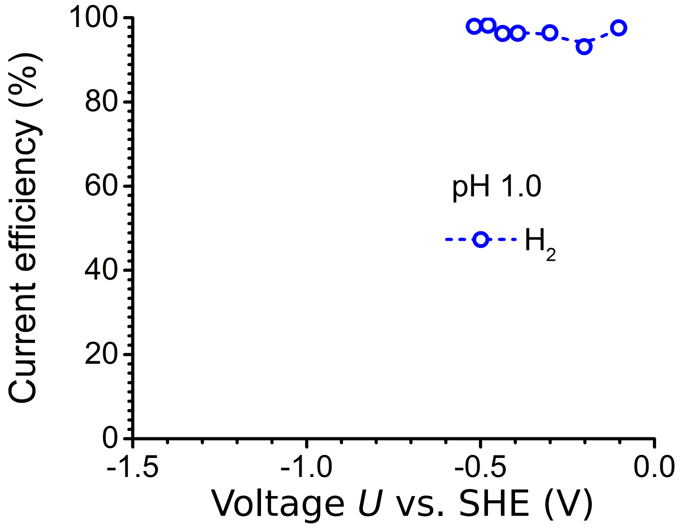

Supplementary Figure 1: Faradaic efficiency for HER under CO_2R pH 1 reaction conditions.

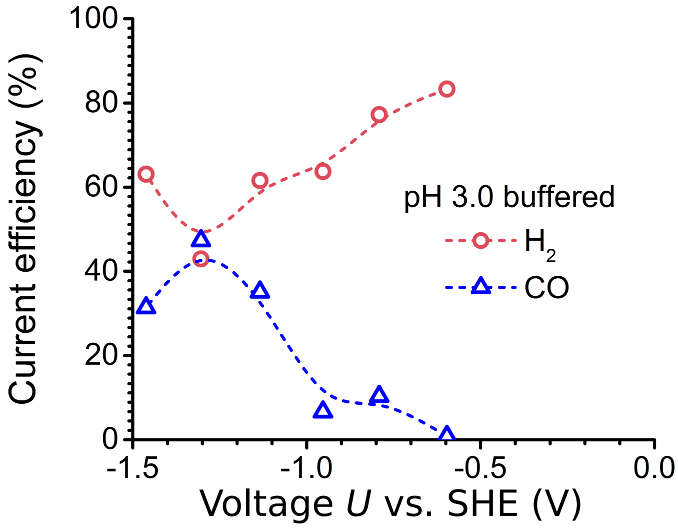

Supplementary Figure 2: Faradaic efficiency for CO and H_2 production under buffered CO_2R pH 3 reaction conditions.

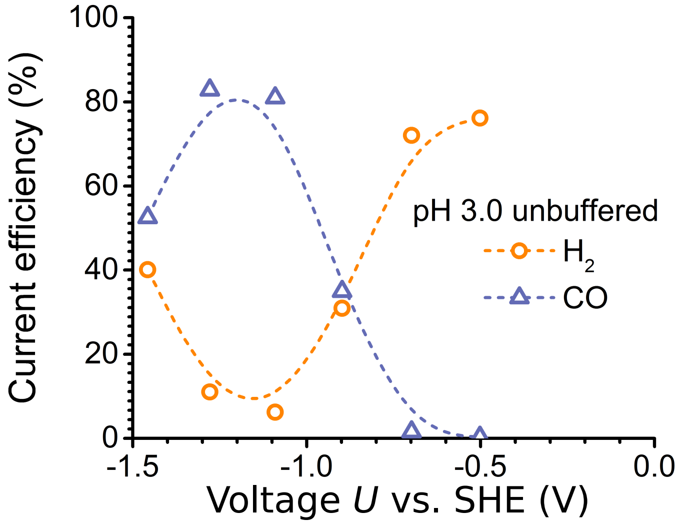

Supplementary Figure 3: Faradaic efficiency for CO and H_2 production under unbuffered CO_2R pH 3 reaction conditions.

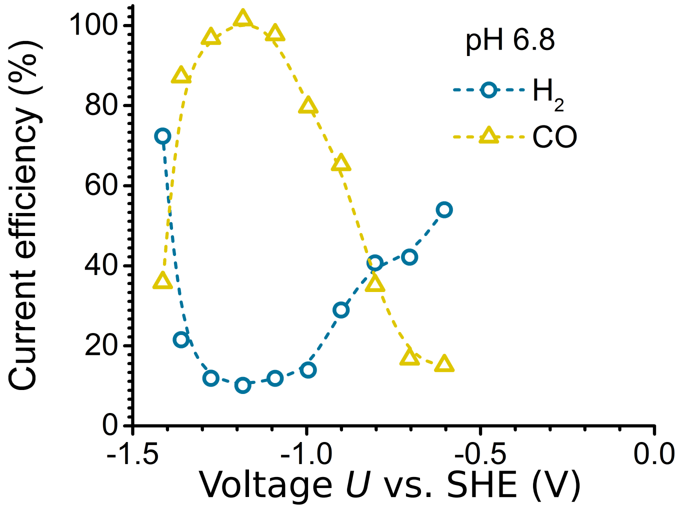

Supplementary Figure 4: Faradaic efficiency for CO and H_2 production under CO_2R pH 6.8 reaction conditions.

Supplementary Note 3: Kinetic barriers & isotope effect

$*CO_2$ to *COOH kinetic barrier. The Nudged Elastic Band (NEB) method was used to calculate the kinetic barrier for $*CO_2$ to *COOH using a total number of seven images. **Supplementary Figure 5**, left, shows the NEB evaluated pathway from $*CO_2$ to *COOH. **Supplementary Figure 5**, right, shows the resulting $*CO_2$ to *COOH barrier extrapolated to a constant electrode potential by correcting for the work function change using a plate-capacitor based approach as described before.[6,7] As seen from the figure, the kinetic barrier is equivalent to the thermochemical barrier, i.e. the transition state behaves electrochemically like the final state.

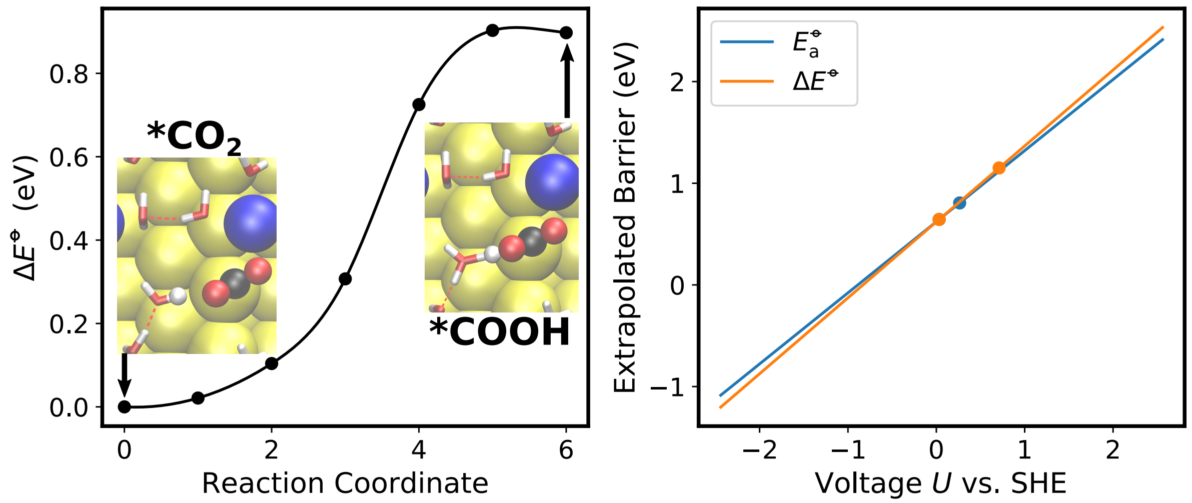

Supplementary Figure 5: Kinetic barrier for the $*CO_2$ to *COOH electrochemical step. *Left*: Nudged Elastic Band derived constant charge kinetic barrier for the $*CO_2$ to *COOH step. *Right*: Extrapolation of kinetic barrier E_a and free energy difference ΔE to the constant electrode potential limit.

Concerted $CO_{2(g)}$ to *COOH kinetic barrier. The Nudged Elastic Band (NEB) method was used to calculate the kinetic barrier for CO_2 in the Helmholtz layer to *COOH using a total number of nine images. **Supplementary Figure 6** shows the resulting pathway. As seen from the figure, the most favorable pathways goes via an adsorbed $*CO_2$ state.

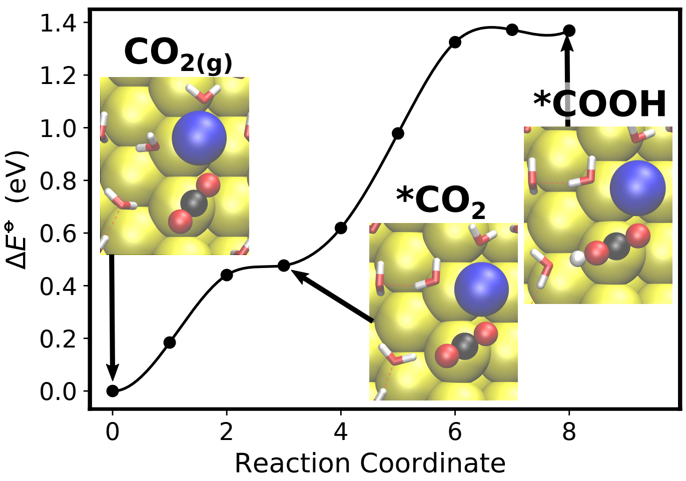

Supplementary Figure 6: Nudged Elastic Band derived constant charge kinetic barrier for the $CO_{2(g)}$ to *COOH step, going via $*CO_2$.

Kinetic isotope effect. The KIE for hydrogen and deuterium is dominated by the zero-point energy (ZPE) E_{ZPE} in the partition function:[8]

$$\frac{k_{\mathrm{H}}}{k_{\mathrm{D}}} \cong \exp\left(-\frac{(E_{\mathrm{ZPE}}^{\mathrm{TS,H}} - E_{\mathrm{ZPE}}^{\mathrm{IS,H}}) - (E_{\mathrm{ZPE}}^{\mathrm{TS,D}} - E_{\mathrm{ZPE}}^{\mathrm{IS,H}})}{RT}\right) \quad .$$

To determine the ZPEs, we carried out a vibrational mode analysis using the harmonic approximation on the initial (IS) and transition (TS) states that we found using NEB-DFT calculations. DFT calculations were performed with a periodic plane-wave implementation and ultrasoft pseudopotentials using QUANTUM ESPRESSO version 5.1[9] interfaced with the Atomistic Simulation Environment (ASE).[10] We applied ultra-soft pseudopotentials and the BEEF-vdW functional, which provides a reasonable description of van der Waals forces while maintaining an accurate prediction of chemisorption energies.[11] Spin-unpolarized calculations were carried out using plane-wave and density cutoffs of 500 and 5000 eV, respectively, as well as a Fermi-level smearing width of 0.1 eV. In general, adsorption energies were evaluated using three-layer 3×1 Au(211) supercells with all but the top layer constrained, 20 Å separation of the surface slabs, 5 water molecules and a single Na^+ cation and 4×4×1 Monkhorst-Pack k-point grids.[12]

The resulting KIE values are summarized in Table **Supplementary Table 5**.

Supplementary Table 5: Kinetic isotope effect (KIE) on two PCET reaction steps of CO_2R using water as a proton donor evaluated from the zero point energies of transition and initial states. Different atoms were vibrated in the case of the first step to demonstrate the expected error range of the results.

Reaction Step	Atoms Vibrated	KIE
$^*CO_2 + H_2O + e^- \rightarrow {}^*COOH + OH^-$	$^*CO_2$ and H	4.6
$^*CO_2 + H_2O + e^- \rightarrow {}^*COOH + OH^-$	$^*CO_2$ and H_2O	3.6
$^*COOH + H_2O + e^- \rightarrow {}^*CO + OH^-$	*COOH	1.2

Supplementary Note 4: Surface charge density dependencies

Supplementary Table 6 and **Supplementary Figure 8** to **Supplementary Figure 10** show the obtained parameters and dependence of the intermediate formation energies on the surface charge density for various facets and different parameter settings relative to the bare slab.

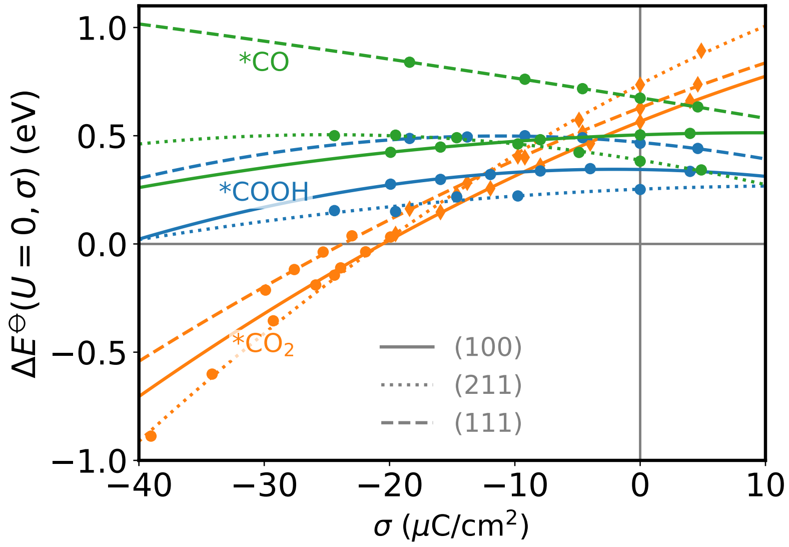

Supplementary Figure 7: Surface charge density dependence of the all reaction intermediates. The BEEF-vdW xc functional was employed as well as the fitPt[13] solvation parameter set. The results are shown for the (211), (111) and the (100) facets.

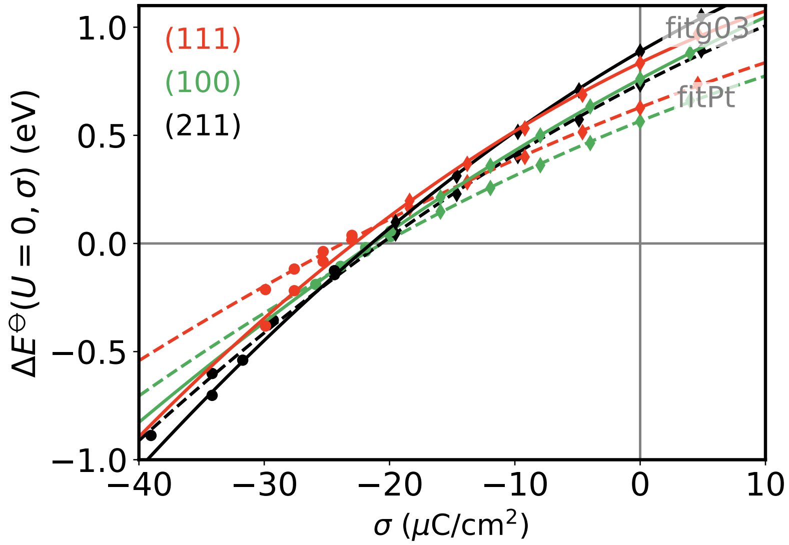

Supplementary Figure 8: Surface charge density dependence of the $*CO_2$ binding energy. The BEEF-vdW xc functional was employed as well as the fitPt[13] and fitg03[14] solvation parameter sets. The results are shown for the (211), (111) and the (100) facets.

Supplementary Table 6: Parabolic fits of the surface charge dependence of the intermediate formation energies relative to the bare slab $\Delta E_m^{\ominus}(U=0,\sigma) = E_m^{\ominus}(U=0,\sigma) - E_0^{\ominus}(U=0,\sigma) = \Delta E_m^{\ominus}(U=0,\sigma=0) + \Delta a_{\sigma,m}\sigma + \Delta b_{\sigma,m}\sigma^2$. *COOH was referenced to $CO_{2,(g)}$ and $H_{2,(g)}$, all remaining adsorbates to their gas phase equivalent. †: The results were obtained with a smaller plane-wave and density cutoffs of 500 and 5000 eV, respectively.

**adsorbate**	**facet**	**unit cell**	**xc functional**	**solvent parameter**	$\boldsymbol{\varepsilon_b}$ ($\boldsymbol{\varepsilon_0}$)	$\boldsymbol{\Delta E_m^{\circ}}$ **(eV)**	$\boldsymbol{\Delta a_{\sigma,m}}$ **($\mathbf{10^{-2}}$ [cm²/μC/eV])**	$\boldsymbol{\Delta b_{\sigma,m}}$ **($\mathbf{10^{-4}}$[cm²/μC/eV]²)**
*CO_2	(111)	3x3	BEEF-vdW	fitg03	6.0	0.84	2.78	-3.86
*CO_2	(111)	3x3	BEEF-vdW	fitPt	6.0	0.63	2.25	-1.71
*CO_2	(100)	3x3	BEEF-vdW	fitg03	6.0	0.76	3.09	-2.20
*CO_2	(100)	3x3	BEEF-vdW	fitPt	6.0	0.57	2.31	-2.18
*CO_2	(211)	3x3	BEEF-vdW	fitg03	6.0	0.89	3.35	-3.69
*CO_2	(211)	3x3	BEEF-vdW	fitPt	6.0	0.74	2.98	-2.87
*CO_2	(211)	3x3	RPBE	fitPt	6.0	0.92	2.23	-6.08
*$CO_2^{\dagger}$	(111)	2x2	BEEF-vdW	fitg03	6.0	0.61	1.59	-2.20
*$CO_2^{\dagger}$	(111)	3x3	BEEF-vdW	fitg03	6.0	0.77	2.87	-1.08
*$CO_2^{\dagger}$	(111)	4x4	BEEF-vdW	fitg03	6.0	0.50	1.05	-5.67
*COOH	(111)	3x3	BEEF-vdW	fitPt	6.0	0.47	-0.53	-2.36
*COOH	(100)	3x3	BEEF-vdW	fitPt	6.0	0.35	-0.09	-2.24
*COOH	(211)	3x3	BEEF-vdW	fitPt	6.0	0.25	0.23	-0.87
*CO	(111)	3x3	BEEF-vdW	fitPt	6.0	0.68	-0.93	-0.20
*CO	(100)	3x3	BEEF-vdW	fitPt	6.0	0.50	0.20	-1.03
*CO	(211)	3x3	BEEF-vdW	fitPt	6.0	0.38	-0.94	-1.89

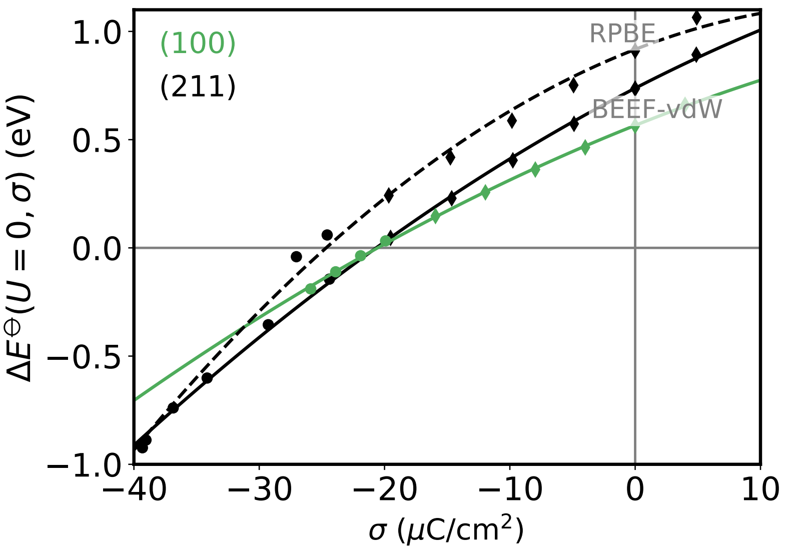

Supplementary Figure 9: Surface charge density dependence of the *CO_2 binding energy. The BEEF-vdW and RPBE xc functionals were employed as well as the fitPt solvation parameter set. The results are shown for the (211) and the (100) facets.

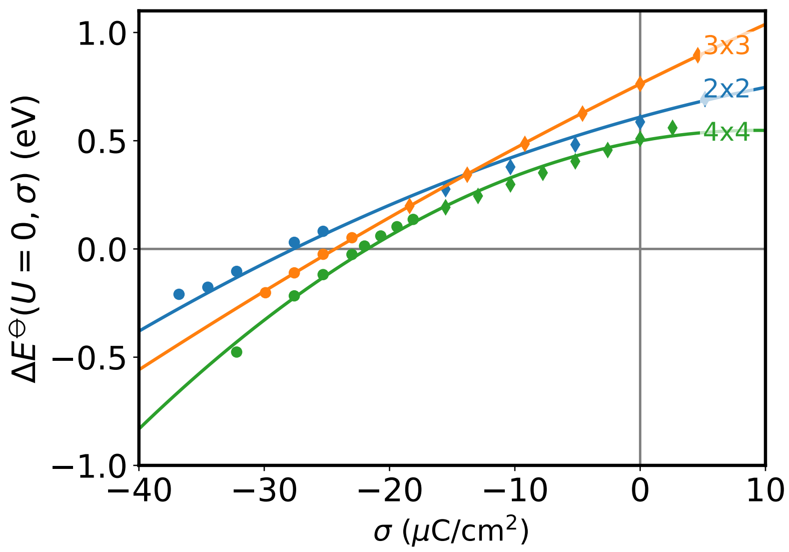

Supplementary Figure 10: Surface charge density dependence of the *CO_2 binding energy. The BEEF-vdW xc functional was employed as well as the fitg03 solvation parameter set for different unit cell sizes. As can be seen from this picture, the unit cell size dependence is small indicating a minor effect of the work function change on the energetics evaluated in the constant charge limit.

Supplementary Note 5: Facet preference of CO_2R

Supplementary Figure 8 shows the surface charge density dependence of all reaction intermediates at the (211), (100) and (111) facets. The Figures shows that $*CO_2$ is stabilized at both the (211) and (100) facet relative to the (111) facet by at least 0.1 eV. **Supplementary Figure 11** further shows that these energy differences result in an order of magnitude smaller activity of the (111) facet compared to the (211) facet. Similar trends have been found before for *COOH showing an energy difference of around 0.25 eV (cf. also ref.[15]) between (211) steps and (111) facet. While these results are partly in line with the preference of under-coordinated sites, they would still enable the reaction to also happen over the terrace sites. In addition, the (100) facet would exhibit similar activity as the step sites. We thus suppose that second order electrolyte effects, such as enhanced water structuring at surface steps,[16,17] the in consequence reduced dielectric screening or specific cation interactions with steps could further increase the activity of under-coordinated sites relative to terraces in addition to our model prediction. Future studies will focus particularly on these explicit electrolyte effects which are beyond the scope of the current work.

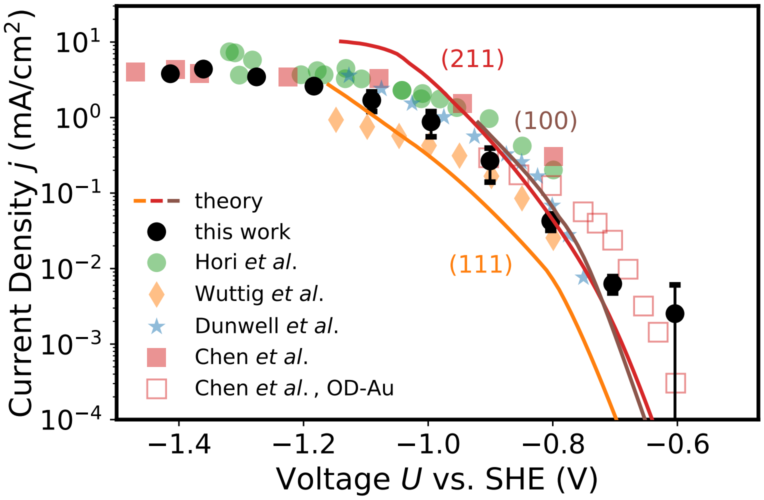

Supplementary Figure 11: `CatINT` simulated CO partial current density. A bulk pH of 6.8 was used and (111), (100) and (211) facets considered for the reaction energies. The theoretical predictions are compared to the experimental results of this work (filled circles).

Supplementary Note 6: Analytic Tafel slope expressions

Starting from the rate-limiting expression for the CO production rate in the case of CO_2 adsorption being limiting:

$$r_1 = a^{\ddagger}_{CO_2}\theta_* e^{-\frac{G^{\ominus}_{*CO_2} - G^{\ominus}_{CO_{2(g)}} - G^{\ominus}_{*}}{RT}} = a^{\ddagger}_{CO_2}\theta_* e^{-\frac{\Delta G^{\ominus}_1(U(\sigma=0)) + \Delta a_{\sigma,1}\sigma(U) + \Delta b_{\sigma,1}\sigma^2(U)}{RT}} \quad \text{(Supplementary Eq. 14)}$$

The effective Tafel slope for CO_2 adsorption as presented in the main paper was obtained by applying the chain rule to the rate expression:

$$
\begin{aligned}
A_{\text{Tafel},1} &= \left|\frac{d\log_{10}(j_{\text{CO}})}{dU}\right|^{-1} \\
&= \left|\frac{\partial\log_{10}(j_{\text{CO}})}{\partial\log_{10}(\theta_*)}\frac{d\log_{10}(\theta_*)}{dU} + \frac{\partial\log_{10}(j_{\text{CO}})}{\partial\Delta G_1^{\ominus}}\frac{d\Delta G_1^{\ominus}}{dU} + \frac{\partial\log_{10}(j_{\text{CO}})}{\partial\log_{10}(a^{\ddagger}_{\text{CO}_2})}\frac{d\log_{10}(a^{\ddagger}_{\text{CO}_2})}{dU}\right|^{-1} \\
&= \left|\underbrace{\frac{d\log_{10}(\theta_*)}{dU}}_{\approx 0} - \log_{10}(e)\frac{\Delta a_{\sigma,1} + 2\Delta b_{\sigma,1}\sigma}{RT}\underbrace{\frac{\partial\sigma}{\partial U}}_{C_{\text{dl}}} + \frac{d\log_{10}(a^{\ddagger}_{\text{CO}_2})}{dU}\right|^{-1} . \qquad \text{(Supplementary Eq. 15)}
\end{aligned}
$$

The first term is close to zero, since the coverages of all species are close to 0. For an arbitrary electrochemical reaction step, and neglecting mass transport effects, the Tafel slope expression becomes:

$$
A_{\text{Tafel},m} = \left|-\log_{10}(e)\left[\frac{\Delta a_{\sigma,m} + 2\Delta b_{\sigma,m}\sigma}{RT}C_{\text{dl}} + (n+\beta)\frac{F}{RT}\right]\right| , \qquad \text{(Supplementary Eq. 16)}
$$

where n is the number of preceding full electron transfers (e.g. PCET). The n comes from the dependence of the activity quotients Q on the potential (cf. Supplementary Note 1), while the β comes from the activation energy. In our case, we use $\beta = 1$ for the $*CO_2$ to *COOH and 0.5 for the *COOH to *CO step. The last term represents the commonly discussed Tafel slope expression (last term in the equation) which is altered by the charge dependence relative to the bare slab (second term).

Supplementary Note 7: Results from multi-scale modeling

Supplementary Figure 12 to **Supplementary Figure 15** show further results from the `CatINT` coupled micro-kinetic-mass transport simulation of CO_2R at Au. In particular, **Supplementary Figure 18** shows the effect of the double layer on water self-dissociation. We find the pK_w to be increased relative to bulk water, i.e. the product of proton and hydroxide concentrations to be decreased, suggesting that less water is in its dissociated form at the double layer. Comparing the hydroxide and hydronium concentration profiles, we find that this is due to the hydronium concentration increasing at a slower rate than the increase in the negative surface charge density, whereas the hydroxide anion concentration decreases at the same rate as the negative surface charge density increases. The reason is the high concentration of potassium cations which hinders the adsorption of hydronium cations by repulsive interactions. Consequently, cations with a smaller size decrease this effect. Interestingly, as shown in **Supplementary Figure 19**, we find the product of the proton and hydroxide activities to be decreased to 12.5 at the reaction plane and -0.9 V vs. SHE. The reason is the increased activity coefficient which destabilizes protons and hydroxide ions, while we assumed that it does not affect water. The interactions with potassium therefore lead to a higher activity of protons and hydroxide ions at the reaction plane. It should be noted that these considerations ignore changes in the kinetics of the water self-dissociation process. Previous *ab initio* studies have suggested e.g. that increased electric fields generally lead to a stronger driving force for water to dissociate.[18] Future studies could incorporate such effects by making the water self-dissociation reaction rates field-dependent.

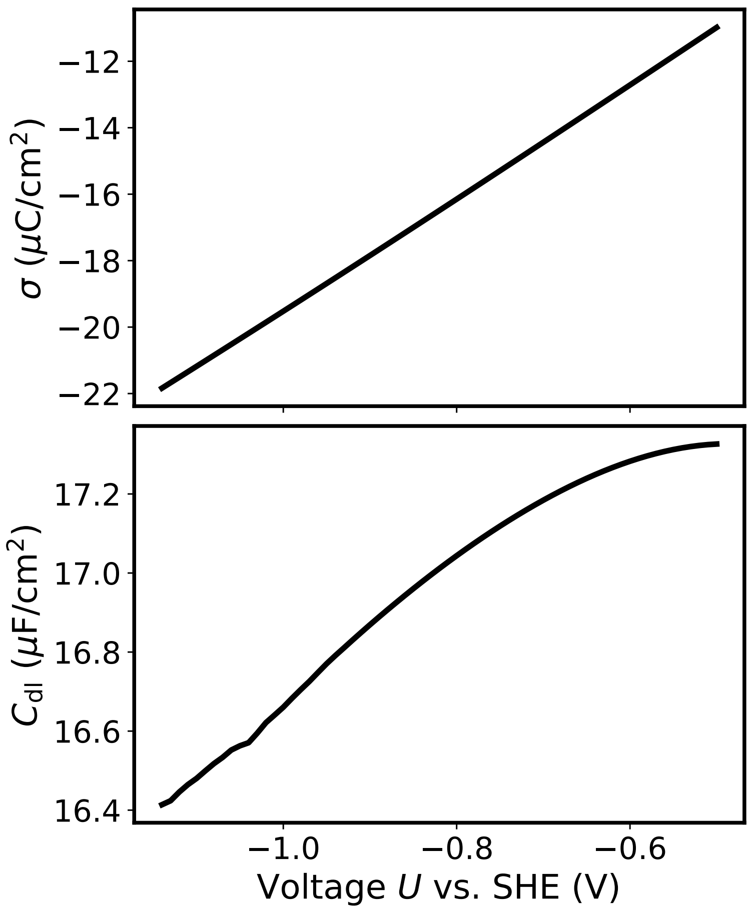

Supplementary Figure 12: Simulated surface charging properties. Surface charge density and double layer capacitance $C_{\mathrm{dl}} = \frac{\partial \sigma}{\partial \phi^{\mathrm{M}}}$ as a function of electrode potential obtained from the `CatINT` simulation at bulk pH of 6.8.

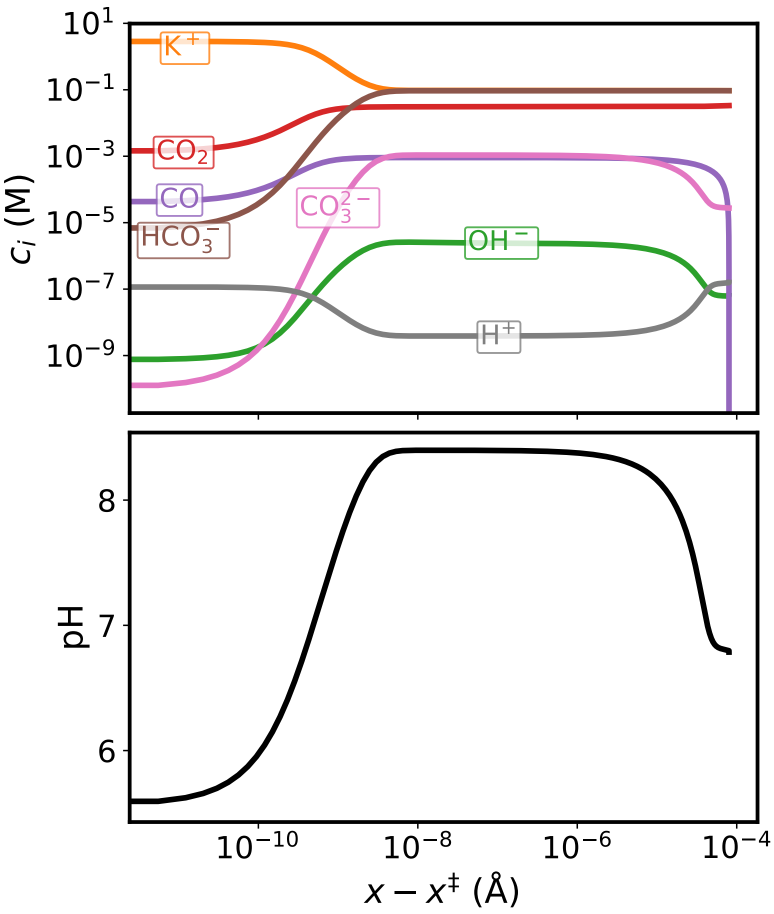

Supplementary Figure 13: Spatial species and pH distribution. Species concentrations (top) and pH (bottom) at -0.9 V vs. SHE as a function of distance from the reaction plane as obtained from the `CatINT` simulation at bulk pH of 6.8.

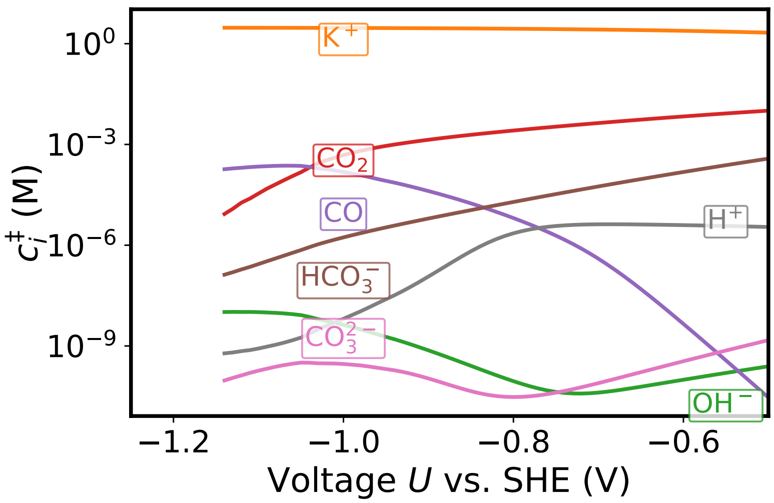

Supplementary Figure 14: Voltage-dependent species concentrations from the `CatINT` simulation at bulk pH of 6.8.

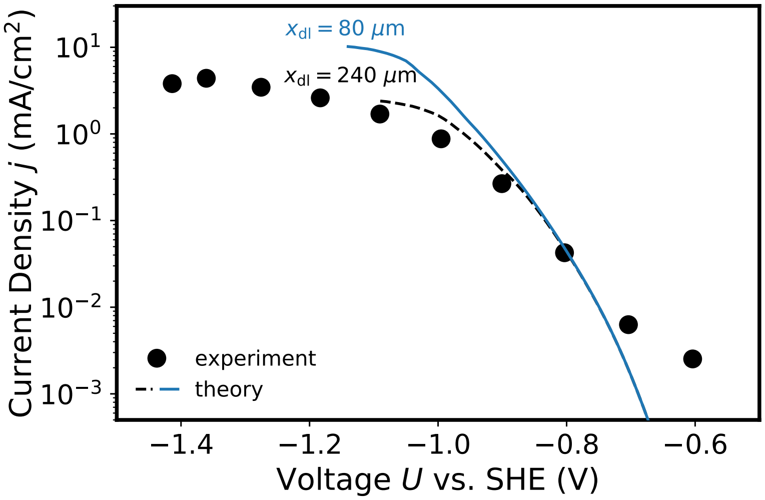

Supplementary Figure 15: Simulated boundary layer thickness dependence of the CO partial current density. A bulk pH of 6.8 was considered with two different boundary layer thicknesses $x_{\rm dl}$. The theoretical predictions are compared to the experimental results of this work (filled circles).

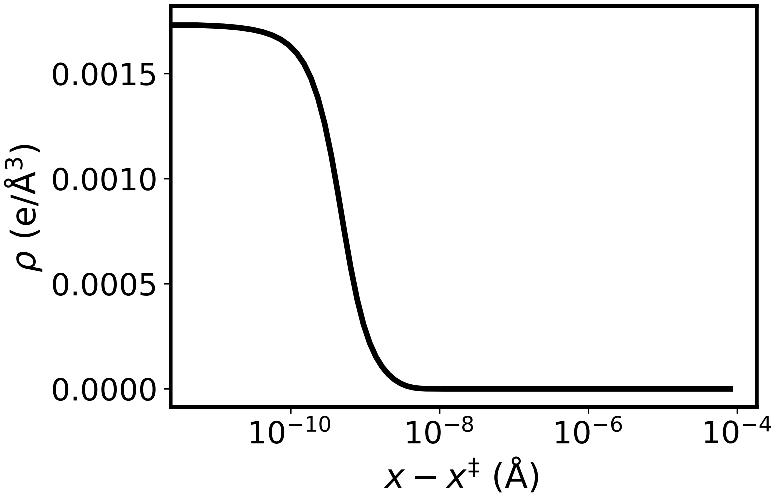

Supplementary Figure 16: Simulated distribution of the charge density. The charge density ρ is given at the reaction plane at a bulk pH of 6.8 and -0.9 V vs. SHE applied voltage.

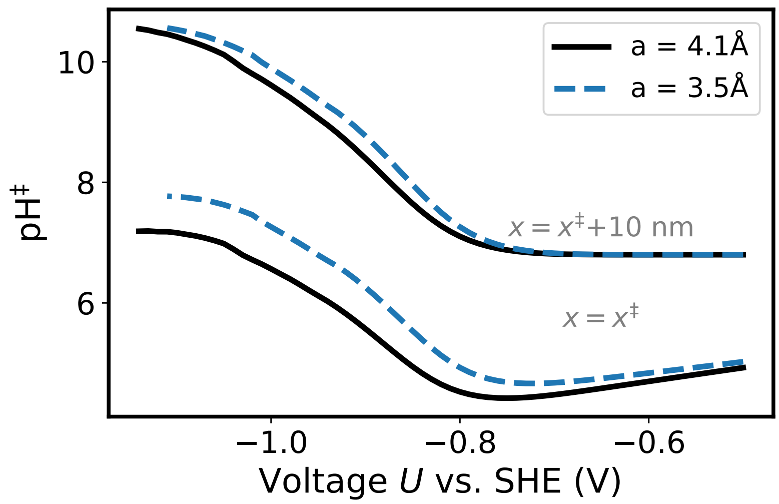

Supplementary Figure 17: Cation size dependence of the local pH. pH at the reaction plane and ENP (10 nm distance from reaction plane) as a function of electrode potential as obtained from the CatINT simulation at bulk pH of 7.0 for two different potassium cation sizes

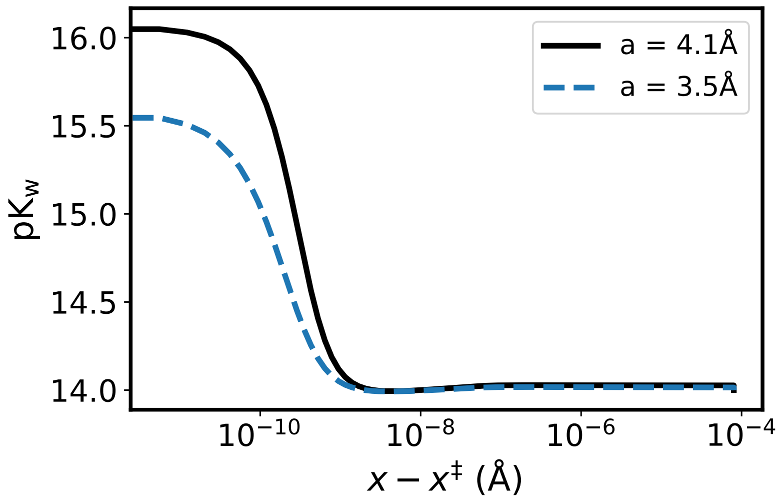

Supplementary Figure 18: Cation size dependence of the pK_w value. The $pK_w = -\log_{10}(c_{H^+} \cdot c_{OH^-}/(1M)^2)$ is given as a function of distance from the reaction plane as obtained from the CatINT simulation at bulk pH of 7.0 for two different potassium cation sizes at -0.9 V vs. SHE.

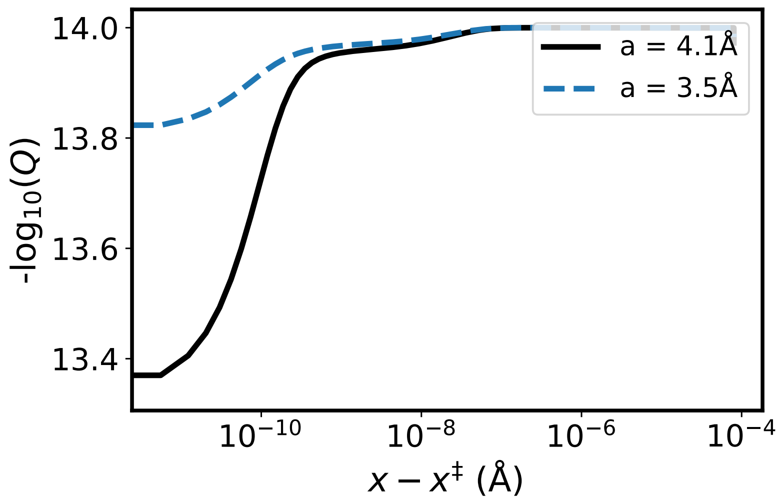

Supplementary Figure 19: Cation size dependence of the water dissociation quotient. The negative decadic logarithm of the water dissociation activity quotient $Q = a_{\mathrm{H}^+} \cdot a_{\mathrm{OH}^-}$ is given as a function of distance from the reaction plane as obtained from the `CatINT` simulation at bulk pH of 7.0 for two different potassium cation sizes at -0.9 V vs. SHE.

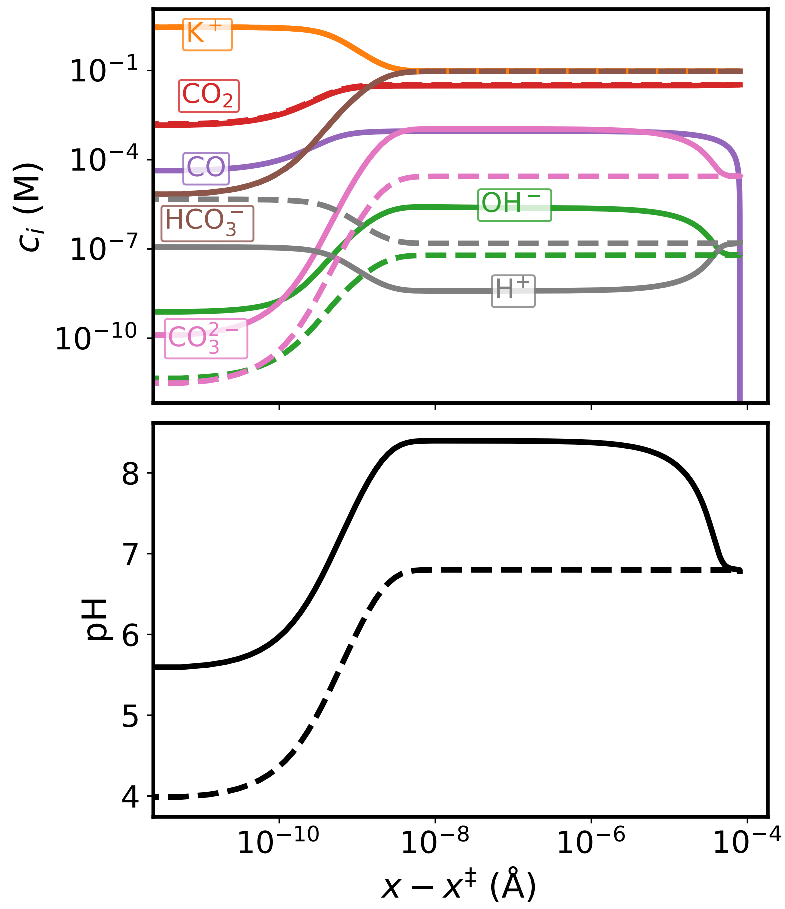

Supplementary Figure 20: Species concentrations (top) and pH (bottom) at -0.9 V vs. SHE as a function of distance from the reaction plane. The profiles were obtained from a `CatINT` simulation at bulk pH of 6.8 with (solid) and without (dashed, zero fluxes) considering electrode reactions.

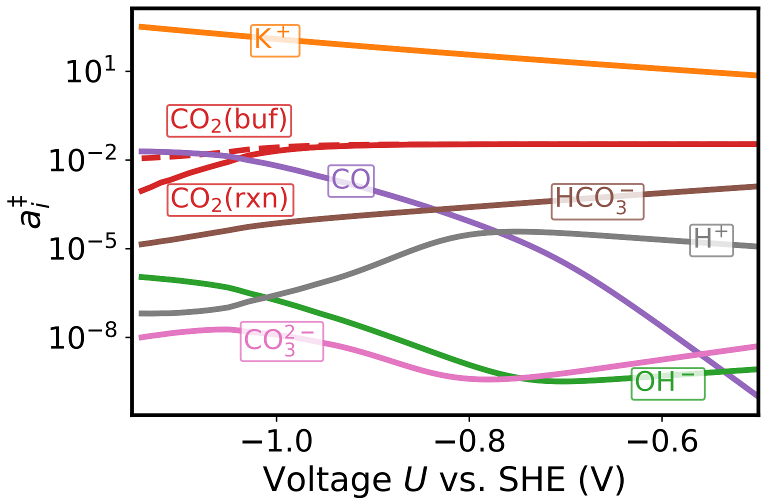

Supplementary Figure 21: Isolated/labeled CO_2 simulations. Species activities at the reaction plane as obtained from the `CatINT` simulation at bulk pH of 6.8 using two different CO_2 species, one participating in the electrode reactions ("rxn") and one in the buffer reactions ("buf").

Supplementary Note 8: Experimental cation effects

CO_2 reduction experiments were carried out using Cs^+ and K^+ electrolyte-containing cations. The results are presented in **Supplementary Figure 22**.

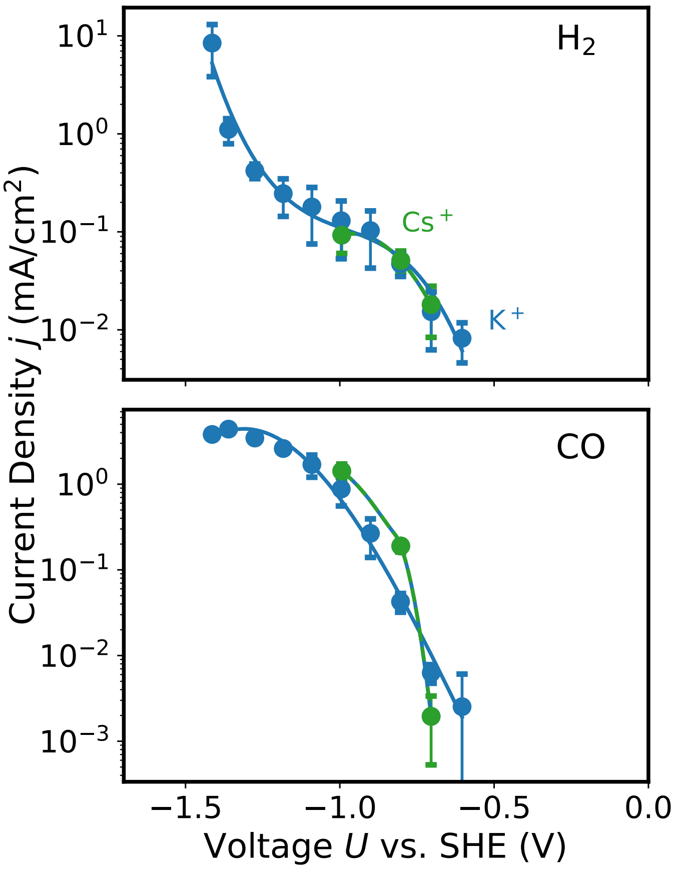

Supplementary Figure 22: Cation effects on the CO and H_2 partial current densities. The experiments were performed in $CsHCO_3$ and $KHCO_3$ electrolytes at pH 6.8 reaction conditions.

Supplementary Methods

Density Functional Theory Calculations. This section describes the Density Functional Theory (DFT) settings employed for the surface charge density dependent calculations that were also used for the micro-kinetic model. For the kinetic barrier and isotope calculations, the reader is referred to Supplementary Note 3. Density functional theory calculations of reaction energetics were carried out with a periodic plane-wave implementation and ultrasoft pseudopotentials using QUANTUM ESPRESSO version 6.1[9] interfaced with the Atomistic Simulation Environment (ASE).[10] We applied ultra-soft pseudopotentials and the BEEF-vdW functional, which provides a reasonable description of van der Waals forces while maintaining an accurate prediction of chemisorption energies.[11] The RPBE[19] functional was also used for testing purposes to quantify the dependence of the results on the choice of the xc functional. Spin-unpolarized calculations were performed using plane-wave and density cutoffs of 700 and 7000 eV, respectively, as well as a Fermi-level smearing width of 0.1 eV. In general, adsorption energies were evaluated using symmetric (adsorbates on both sides of the slab) four-layer 3×3 supercells with all but a single outer layer on both sides constrained, 20 Å separation of the surface slabs, and 4×4×1 Monkhorst-Pack k-point grids.[12] In the case of the (100) surface, we used

five metal layers.

The SCCS implicit solvation model as implemented in the Environ QUANTUM ESPRESSO module[14] was used to model the presence of implicit water. The "fitg03" (in a.u.: $\rho^{\min} = 0.0001$, $\rho^{\max} = 0.005$) solvation parameter set is the default set that has been optimized for neutral molecule solvation energies.[14] Recently, a more accurate parameter set was suggested for properties of metal surfaces by optimizing to the the PZC and capacitance of metal (in particular Platinum) surfaces which we name here the "fitPt" (in a.u.: $\rho^{\min} = 0.0013$, $\rho^{\max} = 0.01025$) parameter set.[13] We decided to use this set, since it is more optimized to surface properties. The bulk dielectric permittivity was set to $\varepsilon_{\mathrm{b}} = 6\varepsilon_0$ (vacuum permittivity ε_0) corresponding to highly constrained water that has been observed at various metal surfaces.[20–25] Cavitation and repulsive energy terms are included by introducing an energy term proportional to the cavity surface area as described in ref.[14] and we here apply the parameter $(\alpha + \gamma) = 11.5\,\mathrm{dyn/cm}$ from the fitg03 parameter set. Dispersion interactions are ignored since they depend on the cavity volume which is an ill-defined property in surface slab calculations. The surface charge density was modulated in order to simulate the response of adsorbate free energies to the presence of an electric double layer field. A planar counter charge with a slab separation of 5 Å was applied to neutralize the simulation cell. A parabolic correction was applied in the Environ calculations to decouple the electrostatic interaction between the periodically repeated slabs. The dependence of each state on the surface charge density was fitted by a parabolic function to obtain the parameters $a_{\sigma,m}$ and $b_{\sigma,m}$ which are used in the kinetic model.

Finally, we note that expressing reaction energies as a function of surface charge density is only exact in the limits of large unit cell sizes. The reason is that the use of finite unit cell sizes induces work function changes during the reaction process while experiments are carried out under electrode potential control. By testing larger unit cell sizes for which the work function change vanishes, we, however, found that the $a_{\sigma,m}$ and $b_{\sigma,m}$ parameters do not critically change while the absolute value of the binding energy deviates by around 100 meV from the cell extrapolated value (cf. **Supplementary Figure 10**).

All structures were relaxed at the corresponding surface charge density using a BFGS line search algorithm until force components were less than 0.03 eV/Å. $*CO_2$ could only be stabilized with at least -1 e surface charge corresponding to around -20 $\mu\mathrm{C/cm^2}$. We therefore extrapolated the formation energy for smaller surface charge densities by performing single point calculations using the optimized geometry at -1 e surface charge/unit cell.

Zero-point energy and finite temperature corrections in the harmonic oscillator approximation were evaluated from the adsorbate vibrations at zero surface charge density using ASE. For the case of $*CO_2$, we used the optimized structure at -1 e of surface charge. We further applied an energy correction of 0.33 eV to the energy of $CO_{2,(g)}$ as well as 0.09 eV to the energy of $H_{2(g)}$ which were determined from fits to experimental gas phase reaction energetics.[11]. In order to evaluate the energy of $H_2O_{(l)}$ from the gas phase formation energy at $P^\circ = 1\,\mathrm{bar}$ (standard reference in `CatMAP`), we used the liquid-vapour equilibrium condition to get an energy correction added on top of the gas phase energy:

$$\mu_{(l)} - \mu_{(g)}^{P^\circ} = \mu_{(g)}^{P_{\mathrm{vap}}} - \mu_{(g)}^{P^\circ} = RT \ln\left(\frac{P_{\mathrm{vap}}}{P^\circ}\right) = -0.089\,\mathrm{eV} \qquad \text{(Supplementary Eq. 17)}$$

using a water vapour pressure of $P_{\mathrm{vap}} = 3.169\,\mathrm{kPa}$ at 298 K.[26] A hydrogen bond stabilization correction of -0.25 eV, was applied to the *COOH intermediate[27] because simplified effective implicit solvation models often do often underestimate this contribution,[28] in particular when using a low dielectric permittivity of 6 as in this study. Double bond corrections of 0.25 eV were applied for *COOH as suggested by ref.[29] as well as for $*CO_2$.

For *COOH and *CO@Au(211) and Au(100), the bridge binding site was considered which was recently shown to be the preferred adsorption site at negative electric fields.[15] For the (111) facet, we considered the top site instead. As seen from the figure, almost all intermediates are stabilized by the negative surface charge density, the one exception being *CO at the (111) atop site which was explained by recent theoretical studies by a surface dipole inversion.[30] For the *COOH to *CO transition state ("$*CO\text{-}OH^{TS}$" in Figure 5 of the main text), we assumed the same surface charge dependence as for *COOH, so that there are no field effects on the barrier and all the potential dependence is integrated into the charge-transfer coefficient.

Multi-scale modeling code `CatINT` . The developed `CatINT` interface provides an easy way to run simulations in the micro-kinetic modeling package `CatMAP`[31] under influence of transport and electric double layer effects simulated by the finite element package `COMSOL Multiphysics`® .[32] A detailed scheme is given in **Supplementary Figure 23** and all applied parameters for modeling CO_2R at Au that were applied in this study are given in **Supplementary Table 7**. Each `CatINT` simulation is defined by three main input files:

1. the `CatINT` input file (`python`)
2. the `CatMAP` input `mkm` file

3. the CatMAP input energies txt file.

The CatINT input file defines all transport-related specifications of the system, as present species, buffers and electrode reactions. Further the calculator for the flux must be defined which in our case is CatMAP . Alternatively, experimental current densities can be also used which is helpful to empirically derive transport-related properties such as concentration profiles.

The CatMAP input files define the micro-kinetic model including all energies of reaction intermediates, kinetic barriers, species pressures, transfer coefficients and adsorbate interactions. We included a modification in the CatMAP module which includes the possibility of running surface charge density σ dependent micro-kinetic modeling. σ is thereby specified as an input parameter. Due to their size $^*CO_2$, *COOH and the *COOH to *CO transition state are assumed to cover two active sites, while *CO is allowed to in principle cover all active sites.

The CatINT input file is parsed to the transport class which initializes all variables. It also evaluates missing concentrations in the input by utilizing the buffer equations, Henry's law or bulk charge neutrality as specified in the input. If the flux calculator is not CatINT , the transport class can further calculate remaining fluxes by utilizing the electrode reaction stoichiometrics.

The transport instance including all it's variables is parsed to the multiple units of the CatINT program package. The Calculator class is the main part taking care of the solution of the transport equations. It contains some basic finite difference algorithms, but for high efficiency computing, the COMSOL Multiphysics® finite element package is linked via the COMSOL class wrapper routine. The latter contains a Model routine which defines the desired transport model as e.g. the GMPNP equations. This model is then written to a java input file, compiled and run. Since the GMPNP including flux boundary conditions and buffer reactions are multi-scale and highly non-linear, the solution is non-trivial and requires optimized grid and solver settings. A finite element mesh with a maximum element size of $L_{\text{cell}}/f_{\text{grid}}^{\text{domain}}$ is used where L_{cell} is the length of the boundary layer (simulation cell) and and $f_{\text{grid}}^{\text{domain}} = 100$. The reaction plane boundary was further resolved by a finer grid using $\lambda_{\text{D}}/f_{\text{grid}}^{\text{bound}}$ where λ_{D} is the Debye length and $f_{\text{grid}}^{\text{bound}} = 200$. We use a non-linear, stationary MUMPS solver to solve the GMPNP equations, where we slowly ramp up the species fluxes at the reaction plane within 100 steps. By this we achieve good convergence in almost all cases. The resulting solutions are stored in the comsol_results directories and contain all transport related properties as concentrations, surface charge density, pH, etc.

If the CatMAP calculator is used to evaluate species fluxes, a self-consistent cycle has to be run between COMSOL Multiphysics® and CatMAP . CatMAP solves the micro-kinetic rate equations in the steady-state approximation yielding species consumption and production rates. These rates are plugged into COMSOL Multiphysics® as flux boundary conditions at the reaction plane. COMSOL Multiphysics® then solves the transport equations yielding the concentrations of species at the reaction plane which are in turn required by CatMAP to define the micro-kinetic model. Between the cycles, the convergence of the current densities is checked and solver settings are automatically adapted if bad convergence in either COMSOL Multiphysics® or the self-consistent cycle is observed. Convergence in the CatINT -COMSOL Multiphysics® cycle was defined when all partial current density changes from the last iteration to the new one (in our case only CO) were below $0.008\,\text{mA/cm}^2$. Regarding COMSOL Multiphysics® , convergence can be e.g. improved by decreasing the finite element grid spacing or smoothening the non-linearity ramping of the flux and/or Helmholtz capacitance and PZC. The CatMAP -COMSOL Multiphysics® iterations can further be stabilized by using a mixing factor of $\gamma = 0.02$ resulting in only 3 iterations needed at low overpotentials and up to around 20-30 at higher overpotentials. Results were obtained for potentials starting from -0.5 to -2.0 V vs. SHE using a 0.01 V step interval. At each potential, the solutions for the reaction plane concentrations of species were always initialized from the previous potential step in order to further increase the stability of the solver. At high overpotentials, often convergence problems arose, e.g. due to diminishing CO_2 leading to slightly negative concentrations so that that only a potential range until -1.2 to -1.4 V vs. SHE was accessible in this paper.

Beside the reaction plane species concentrations, also the surface charge density σ is parsed from COMSOL Multiphysics® to CatMAP . This is required to define the free energies as a function of σ using the parabolic σ-dependence derived from DFT.

Parametrization of micro-kinetic and mass transport models. The transport model relies on a couple of macroscopic parameters which have been mostly extracted from literature, as the diffusion coefficients or the Henry constants to estimate the bulk concentration of gaseous species. All constants have been collected in **Supplementary Table 7**. The boundary layer thickness x_{bl} was set to a standard value of $80\,\mu$m that we found in a previous publication[33] and also again in this study. The value is only slightly dependent on the electrode and system conditions and as **Supplementary Figure 15** shows the results depend only slightly on the exact value. The active site density was taken from the density of grain boundaries as described in the main text. We further used a pre-exponential factor of 10^{13}s^{-1} that was chosen based on the entropy-free pre-factor $k_{\text{B}}T/h$ (Boltzmann constant k_{B}) that appears in transition state theory[34] which was used by various kinetic CO_2R studies.[29,35] Experimentally, this value has been

confirmed for CO desorption,[36] and a similar magnitude $10^{11} - 10^{13} s^{-1}$ for CO_2 desorption.[37]

As also written in the main text, we then set the electrostatic parameters (parameter set II in **Supplementary Table 7**) $C_{gap} = 20\,\mu F/cm^2$[38–40] and $\Delta\phi^{M,PZC} = 0.16\,V$ vs. SHE (PZC measured in NaF aqueous solutions[41]). The cation radius of the supporting cation K^+ was set to 4.1 Å being the distance of K^+ cations from Ag surfaces measured with x-ray diffraction techniques at negative overpotentials.[42] The value of the size is quite important since it determines the charging of the surface and therefore the field stabilization of the reaction intermediates. Further a non-zero cation size prevents numerical problems arising with high cation concentrations at the highly negatively charged electrode.

Concentrations of all species in the bulk also serve as initialization for the spatially dependent species concentrations in the transport model (parameter set IV in **Supplementary Table 7**). The concentration of dissolved CO_2 is estimated from the Henry constant, since CO_2 is constantly bubbled during the experiment leaving the solution with an equilibrium content of CO_2 . The CO concentration was set to zero and concentrations of protons and hyroxide ions are given by the input pH. Concentrations of bicarbonate and carbonate are then pre-determined by either the alkaline (cf. parameter set V a) or acidic (cf. parameter set V b) set of buffer reactions.

Supplementary Table 7: Constants used in the transport model at 25°C and for the bulk pH = 6.8 reaction conditions: Boundary layer thickness x_{bl}, active site area ρ_{act}, Helmholtz capacitance C_H, PZC $\Delta\phi^{M,PZC}$, MPB lattice cell parameter a_i^{cell} (= 2× ion radius), diffusion constants D_i, Henry constants H_i^{cp}, bulk solvent reference concentrations c_i°, equilibrium constants K^{eq} and rate constants $k^{\rightarrow}$. The backward rate constants $k^{\leftarrow}$ were obtained from $k^{\leftarrow} = \frac{k^{\rightarrow}}{K^{eq}}$.

Set	Property (Unit)	Source	Species/Reaction	Value
**I**	x_{bl} (m)	this work	–	$80 \cdot 10^{-5}$
	ρ_{act} (sites/Å^2)	this work and ref.[43]	pc-Au	$9.6 \cdot 10^{-5}$
**II**	C_{gap} ($\mu F/cm^2$)	refs.[38–40]	pc-Au (also other facets and metals)	20
	$\Delta\phi^{M,PZC}$ (V)	ref.[41]	pc-Au	0.16
	a_i^{cell} (Å)	ref.[42]	K^+	8.2
**III**	D_i (m^2/s)	ref.[44]	CO_2	$1.91 \cdot 10^{-9}$
		ref.[44]	CO	$2.23 \cdot 10^{-9}$
		ref.[45]	HCO_3^-	$1.185 \cdot 10^{-9}$
		ref.[45]	CO_3^{2-}	$0.923 \cdot 10^{-9}$
		ref.[45]	OH^-	$5.273 \cdot 10^{-9}$
		ref.[45]	K^+	$1.957 \cdot 10^{-9}$
**IV a)**	H_i^{cp} ($mol/m^3/Pa$)	ref.[46]	CO	$9.7 \cdot 10^{-6}$
		ref.[46]	CO_2	$3.3 \cdot 10^{-4}$
**IV b)**	c_i° (M)	$H_i^{cp} P_i^\circ$	CO_2	0.033
		–	CO	0.0
		10^{pH-14}	OH^-	$6.31 \cdot 10^{-8}$
		10^{-pH}	H^+	$1.58 \cdot 10^{-7}$
		buffer equilibria equations	HCO_3^-	0.091
		buffer equilibria equations	CO_3^{2-}	$2.68 \cdot 10^{-5}$
**V a)**	K^{eq} (M^{-1})	ref.[47]	$CO_2 + OH^- \rightleftharpoons HCO_3^-$	$4.44 \cdot 10^{7}$
	$k^{\rightarrow}$ ($M^{-1}s^{-1}$)	ref.[47]	$CO_2 + OH^- \rightleftharpoons HCO_3^-$	$5.93 \cdot 10^{3}$
	K^{eq} (M^{-1})	ref.[47]	$HCO_3^- + OH^- \rightleftharpoons CO_3^{2-} + H_2O$	$4.66 \cdot 10^{3}$
	$k^{\rightarrow}$ ($M^{-1}s^{-1}$)	ref.[47]	$HCO_3^- + OH^- \rightleftharpoons CO_3^{2-} + H_2O$	$1 \cdot 10^{8}$
**V b)**	K^{eq} (M)	ref.[47]	$CO_2 + H_2O \rightleftharpoons HCO_3^- + H^+$	$4.44 \cdot 10^{-7}$
	$k^{\rightarrow}$ (s^{-1})	ref.[48]	$CO_2 + H_2O \rightleftharpoons HCO_3^- + H^+$	$3.7 \cdot 10^{-2}$
	K^{eq} (M)	from K_w and K^{eq} of alkaline rxn	$HCO_3^- + \rightleftharpoons CO_3^{2-} + H^+$	$4.66 \cdot 10^{-5}$
	$k^{\rightarrow}$ (s^{-1})	ref.[49]	$HCO_3^- + \rightleftharpoons CO_3^{2-} + H^+$	$59.44 \cdot 10^{8}$
**V c)**	K_w (M^2)	ref.[48]	$H_2O \rightleftharpoons H^+ + OH^-$	$1 \cdot 10^{-14}$
	$k^{\rightarrow}$ (Ms^{-1})	ref.[48]	$H_2O \rightleftharpoons H^+ + OH^-$	$2.4 \cdot 10^{-5}$

Chemical reactions in the electrolyte were included by extending previous works (parameter set V).[47,50] In contrast to these references, which either modeled the bicarbonate buffer by reactions in acid or base, we decided to use both the reaction in base (parameter set V a) and in acid (set V b) at the same time. The reason is the neutral pH that we are simulating making both the presence of hydroxide anions and protons nearly equally likely. Additionally, we included the water self-dissociation equilibrium (set V c) which we found to be important for the actual buffering effect.

Concentrations, activities and references. An important question is the definition of the reference for all the energetics that enter both the transport and kinetic model. All energetics that are defined in the `CatMAP` energies `.txt` file are given with reference to 1 bar of partial pressure of the input gas. `CatMAP` supports the setting of a different `pressure` for the species and then recalculates the energetics based on

$$\mu_{(g)}^{P_i} = \mu_{(g)}^{P_i^\circ} + RT \ln\left(\frac{P_i}{P_i^\circ}\right) \quad , \qquad \text{(Supplementary Eq. 18)}$$

with $P_i^\circ = 1\,\text{bar}$. In our case, the reactant CO_2 is dissolved in the liquid and the transport model provides us with an estimate for the local CO_2 activity $a_i = \gamma_i c_i$. We can use Henry's law to estimate the corresponding gas phase pressure which can then enter Eq. Supplementary Eq. 18:

$$P_i = a_i / H_i^{\text{cp}} \quad , \qquad \text{(Supplementary Eq. 19)}$$

and we use the same approach for the gaseous CO product. Note that all pressures and activities given here refer to their values at the reaction plane. Proton and hydroxide activities enter into the kinetic model directly via the pH and the reference electrode (in our case the SHE) defines pH = 0 as the relevant reference.

Kinetic barriers for the chemical reactions in the electrolyte are all given with respect to a 1 M concentrated, but ideal reference solution. Concentrations from our model can be therefore directly plugged into the defining kinetic rate equations as described before,[47,50] but need to be multiplied by the activity coefficients defined by the GMPB model.

Current density. The current density j_i can be obtained from the flux of the respective species i_i via

$$j_i = \xi_e / \xi_i F \rho_{\text{act}} i_i \quad , \qquad \text{(Supplementary Eq. 20)}$$

where ρ_{act} denotes the active site density and ξ_e and ξ_i represent the reaction equivalents of electrons and products in the reaction equation. For CO production, e.g. $\xi_e = 2$ (2 e^--process) and $\xi_{\text{CO}} = 1$.

Double layer capacitance and Robin boundary condition. The Helmholtz gap capacitance can be generally written as

$$C_{\text{gap}} = \frac{\text{d}\sigma}{\text{d}(\phi^{\text{M}} - \phi^{\ddagger})} \quad , \qquad \text{(Supplementary Eq. 21)}$$

where ϕ^{M} denotes the potential inside the metal electrode and $\phi^{\ddagger}$ the potential outside of the gap. Considering a planar electrode gives further rise to a constant electric field within the gap. Using this and integrating C_{gap} in Eq. Supplementary Eq. 21 from the PZC to the metal potential of interest, this then leads to a Robin boundary condition for the electrostatic potential:[51]

$$\begin{aligned}
\sigma &= -\varepsilon_{\text{b}} \left.\frac{d\phi^{\ddagger}}{dx}\right|_{x^{\ddagger}} \\
&= C_{\text{gap}} \left[\left(\phi^{\text{M}} - \phi^{\ddagger}\right) - \left(\phi^{\text{M,PZC}} - \phi^{\ddagger,\text{PZC}}\right)\right] \\
&= C_{\text{gap}} \left[\left(\phi^{\text{M}} - \phi^{\ddagger}\right) - \phi^{\text{M,PZC}}\right] \\
&= C_{\text{gap}} \left[\left(\Delta\phi^{\text{M}} - \phi^{\ddagger}\right) - \Delta\phi^{\text{M,PZC}}\right] \\
&= C_{\text{gap}} \left[\left(U - \phi^{\ddagger}\right) - U^{\text{PZC}}\right] \quad . \qquad \text{(Supplementary Eq. 22)}
\end{aligned}$$

We here used that at the PZC, $\phi^{\ddagger,\text{PZC}} \approx \phi^0$. In the last two lines, we further referenced the potentials to the absolute SHE reference scale, and then included the chemical potential difference between the working and reference electrodes in order to utilize the experimentally accessible PZC U^{PZC}. U^{PZC} is then the measured PZC of the working electrode relative to the reference electrode.

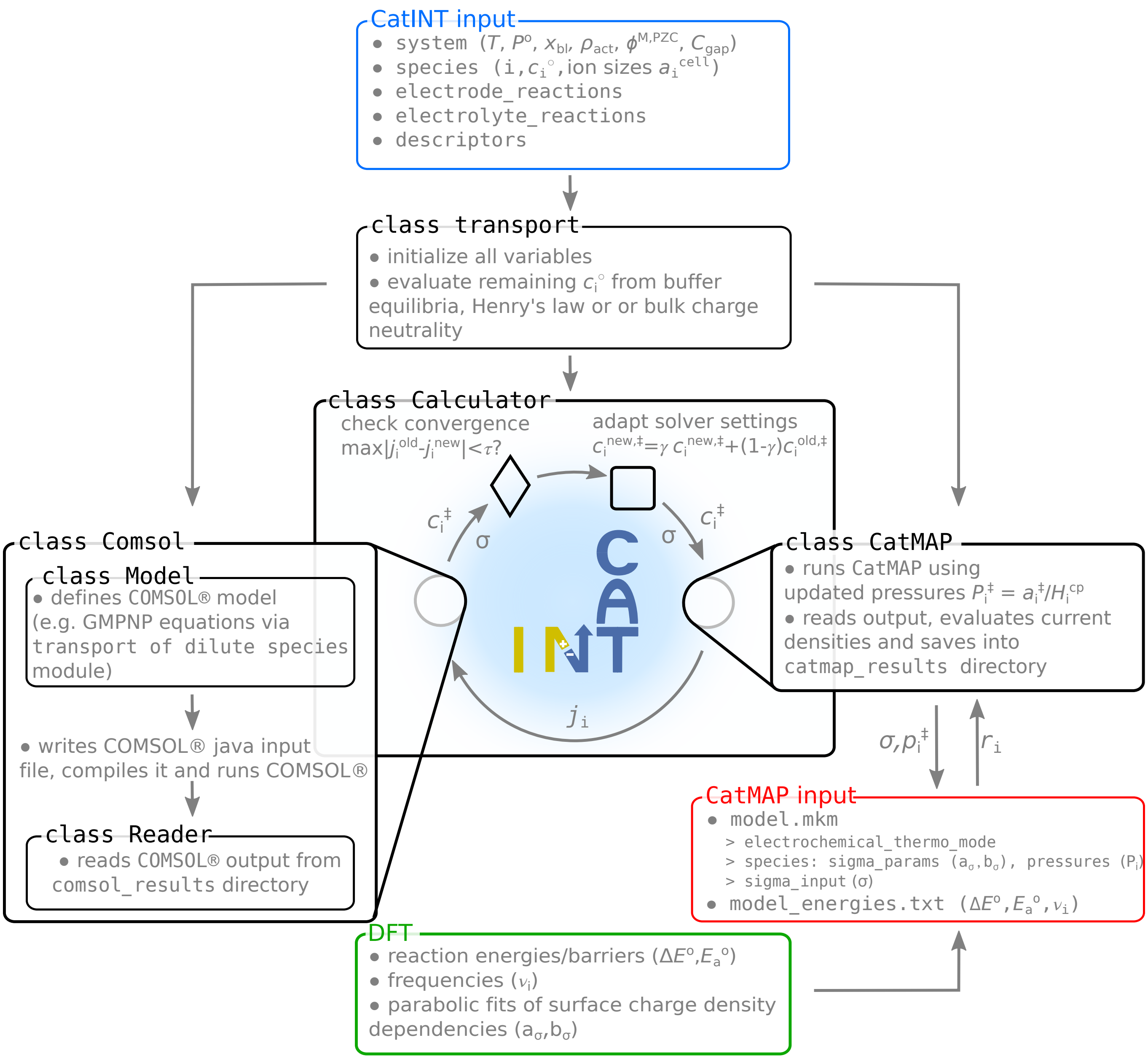

Supplementary Figure 23: Schematic illustration of the `CatINT` software package for multi-scale modeling. In order to set up a coupled micro-kinetic transport model, a `CatINT` input file is required which contains all information about present species, buffer and electrode reactions that need to be simulated. The input is parsed to the `transport` class which initializes and stores all `CatINT` variables. A `calculator` class instance can then be created which solves the coupled micro-kinetic-GMPNP equation system. For that it calls the `CatMAP` class which runs `CatMAP` using the current species concentrations at the reaction plane as well as the surface charge density. The resulting species fluxes are used to set up the flux boundary condition of the `COMSOL Multiphysics`® GMPNP model. The resulting concentrations at the reaction plane are linearly mixed with the results from the previous cycle in order to improve convergence. The `CatMAP` –`COMSOL Multiphysics`® cycle is repeated until convergence in the current densities is achieved.

Supplementary References

[1] Nørskov, J. K. *et al.* Origin of the overpotential for oxygen reduction at a Fuel-Cell cathode. *J. Phys. Chem. B* **108**, 17886–17892 (2004).
[2] Gauthier, J. A., Dickens, C. F., Ringe, S. & Chan, K. Practical considerations for continuum models applied to surface electrochemistry. *Chemphyschem* (2019).
[3] Ringe, S. *et al.* Understanding cation effects in electrochemical CO2 reduction. *Energy Environ. Sci.* (2019).
[4] Gauthier, J., Dickens, C., Heenen, H. H., Ringe, S. & Chan, K. Unified approach to implicit and explicit solvent simulations of electrochemical reaction energetics (2019).
[5] Bazant, M. Z. Theory of chemical kinetics and charge transfer based on nonequilibrium thermodynamics. *Acc. Chem. Res.* **46**, 1144–1160 (2013).
[6] Chan, K. & Nørskov, J. K. Electrochemical barriers made simple. *J. Phys. Chem. Lett.* **6**, 2663–2668 (2015).
[7] Chan, K. & Nørskov, J. K. Potential dependence of electrochemical barriers from ab initio calculations. *J. Phys. Chem. Lett.* **7**, 1686–1690 (2016).
[8] Bigeleisen, J. The relative reaction velocities of isotopic molecules. *J. Chem. Phys.* **17**, 675–678 (1949).
[9] Giannozzi, P. *et al.* QUANTUM ESPRESSO: a modular and open-source software project for quantum simulations of materials. *J. Phys. Condens. Matter* **21**, 395502 (2009).
[10] Hjorth Larsen, A. *et al.* The atomic simulation environment—a python library for working with atoms. *J. Phys. Condens. Matter* **29**, 273002 (2017).
[11] Studt, F., Abild-Pedersen, F., Varley, J. B. & Nørskov, J. K. CO and CO2 hydrogenation to methanol calculated using the BEEF-vdW functional. *Catal. Letters* **143**, 71–73 (2013).
[12] Monkhorst, H. J. & Pack, J. D. Special points for brillouin-zone integrations. *Phys. Rev. B Condens. Matter* **13**, 5188–5192 (1976).
[13] Hörmann, N. G., Andreussi, O. & Marzari, N. Grand canonical simulations of electrochemical interfaces in implicit solvation models. *J. Chem. Phys.* **150**, 041730 (2019).
[14] Andreussi, O., Dabo, I. & Marzari, N. Revised self-consistent continuum solvation in electronic-structure calculations. *J. Chem. Phys.* **136**, 064102 (2012).
[15] Clark, E. L. *et al.* Influence of atomic surface structure on the activity of ag for the electrochemical reduction of CO2 to CO. *ACS Catal.* (2019).
[16] Schwarz, K., Xu, B., Yan, Y. & Sundararaman, R. Partial oxidation of Step-Bound Water Leads to Anomalous pH Effects on Metal Electrode Step-Edges. *Phys. Chem. Chem. Phys.* **18**, 16216–16223 (2016). 1605.00550.
[17] Huzayyin, A., Chang, J. H., Lian, K. & Dawson, F. Interaction of water molecule with au(111) and au(110) surfaces under the influence of an external electric field. *J. Phys. Chem. C* **118**, 3459–3470 (2014).
[18] Saitta, A. M., Saija, F. & Giaquinta, P. V. Ab initio molecular dynamics study of dissociation of water under an electric field. *Phys. Rev. Lett.* **108**, 207801 (2012).
[19] Hammer, B., Hansen, L. B. & Nørskov, J. K. Improved adsorption energetics within density-functional theory using revised Perdew-Burke-Ernzerhof functionals. *Phys. Rev. B Condens. Matter* **59**, 7413–7421 (1999).
[20] Toney, M. F. *et al.* Voltage-dependent ordering of water molecules at an electrode–electrolyte interface. *Nature* **368**, 444 (1994).
[21] Toney, M. F. *et al.* Distribution of water molecules at ag(111)/electrolyte interface as studied with surface x-ray scattering. *Surf. Sci.* **335**, 326–332 (1995).
[22] Parsons, R. The metal-liquid electrolyte interface. *Solid State Ionics* **94**, 91–98 (1997).
[23] Noguchi, H., Okada, T. & Uosaki, K. Molecular structure at electrode/electrolyte solution interfaces related to electrocatalysis. *Faraday Discuss.* **140**, 125–137 (2008).
[24] Beltramo, G., Giesen, M. & Ibach, H. Anomalous helmholtz-capacitance on stepped surfaces of silver and gold. *Electrochim. Acta* **54**, 4305–4311 (2009).
[25] Fumagalli, L. *et al.* Anomalously low dielectric constant of confined water. *Science* **360**, 1339–1342 (2018).
[26] Lide, D. R. *CRC Handbook of Chemistry and Physics, 85th Edition* (CRC Press, 2004).
[27] Peterson, A. A., Abild-Pedersen, F., Studt, F., Rossmeisl, J. & Nørskov, J. K. How copper catalyzes the electroreduction of carbon dioxide into hydrocarbon fuels. *Energy Environ. Sci.* **3**, 1311–1315 (2010).
[28] Cramer, C. J. & Truhlar, D. G. Implicit solvation models: Equilibria, structure, spectra, and dynamics. *Chem. Rev.* **99**, 2161–2200 (1999).
[29] Liu, X. *et al.* ph effects on the electrochemical reduction of CO(2) towards C2 products on stepped copper. *Nat. Commun.* **10**, 32 (2019).
[30] Duan, Z. & Henkelman, G. Calculations of the pH-Dependent onset potential for CO electrooxidation on au(111). *Langmuir* **34**, 15268–15275 (2018).
[31] Medford, A. J. *et al.* CatMAP: A software package for Descriptor-Based microkinetic mapping of catalytic trends. *Catal. Lett.* **145**, 794–807 (2015).
[32] COMSOL multiphysics v. 5.3a, comsol ab, stockholm, sweden. `http://www.comsol.com`.
[33] Morales-Guio, C. G. *et al.* Improved CO2 reduction activity towards c2+ alcohols on a tandem gold on copper electrocatalyst. *Nature Catalysis* **1**, 764–771 (2018).
[34] Nørskov, J. K., Studt, F., Abild-Pedersen, F. & Bligaard, T. *Fundamental Concepts in Heterogeneous Catalysis* (2014).
[35] Hansen, H. A., Varley, J. B., Peterson, A. A. & Nørskov, J. K. Understanding trends in the electrocatalytic activity of metals and enzymes for CO2 reduction to CO. *J. Phys. Chem. Lett.* **4**, 388–392 (2013).
[36] Vollmer, S., Witte, G. & Wöll, C. Determination of site specific adsorption energies of CO on copper. *Catal. Letters* **77**, 97–101 (2001).
[37] Solymosi, F. The bonding, structure and reactions of CO2 adsorbed on clean and promoted metal surfaces. *Journal of Molecular Catalysis* **65**, 337–358 (1991).
[38] Amokrane, S. & Badiali, J. P. Analysis of the capacitance of the Metal-Solution interface: Role of the metal and the Metal-Solvent coupling. In White, R. E. & J. O'M. Bockris, B. E. C. (eds.) *Modern Aspects of Electrochemistry*, vol. 22, 1–95 (Butterworths, New York).

[39]Eberhardt, D., Santos, E. & Schmickler, W. Impedance studies of reconstructed and non-reconstructed gold single crystal surfaces. *J. Electroanal. Chem.* **419**, 23–31 (1996).
[40]Pajkossy, T., Wandlowski, T. & Kolb, D. M. Impedance aspects of anion adsorption on gold single crystal electrodes. *J. Electroanal. Chem.* **414**, 209–220 (1996).
[41]Trasatti, S. & Lust, E. The potential of zero charge. In White, R. E. & J. O'M. Bockris, B. E. C. (eds.) *Modern Aspects of Electrochemistry*, vol. 33 (KluwerAcademic/PlenumPublishers, New York).
[42]Lucas, C. A., Thompson, P., Gründer, Y. & Markovic, N. M. The structure of the electrochemical double layer: Ag(111) in alkaline electrolyte. *Electrochem. commun.* **13**, 1205–1208 (2011).
[43]Mariano, R. G., McKelvey, K., White, H. S. & Kanan, M. W. Selective increase in CO2 electroreduction activity at grain-boundary surface terminations. *Science* **358**, 1187–1192 (2017).
[44]Diffusion of gases in water. In Lide, D. R. (ed.) *CRC Handbook of Chemistry and Physics*, 6–180 (CRC Press LLC).
[45]Van\'ysek, P. Ionic conductivity and diffusion at infinite dilution. In Lide, D. R. (ed.) *CRC Handbook of Chemistry and Physics*, 5–92 (CRC Press LLC).
[46]Sander, R. Compilation of henry's law constants (version 4.0) for water as solvent. *Atmos. Chem. Phys.* **15**, 4399–4981 (2015).
[47]Gupta, N., Gattrell, M. & MacDougall, B. Calculation for the cathode surface concentrations in the electrochemical reduction of CO2 in KHCO3 solutions. *J. Appl. Electrochem.* **36**, 161–172 (2006).
[48]Emerson, S. & Hedges, J. Carbonate chemistry. In *Chemical Oceanography and the Marine Carbon Cycle*, 101–133 (Cambridge University Press, 2008).
[49]Schulz, K. G., Riebesell, U., Rost, B., Thoms, S. & Zeebe, R. E. Determination of the rate constants for the carbon dioxide to bicarbonate inter-conversion in ph-buffered seawater systems. *Mar. Chem.* **100**, 53–65 (2006).
[50]Singh, M. R., Goodpaster, J. D., Weber, A. Z., Head-Gordon, M. & Bell, A. T. Mechanistic insights into electrochemical reduction of CO2 over ag using density functional theory and transport models. *Proc. Natl. Acad. Sci. U. S. A.* **114**, E8812–E8821 (2017).
[51]Chan, K. & Eikerling, M. A Pore-Scale model of oxygen reduction in Ionomer-Free catalyst layers of PEFCs. *J. Electrochem. Soc.* **158**, B18–B28 (2011).
